# Geographic factors and climatic fluctuation drive the genetic structure and demographic history of *Cycas taiwaniana* (Cycadaceae), an endemic endangered species to Hainan Island in China

**DOI:** 10.1002/ece3.9508

**Published:** 2022-11-18

**Authors:** Li‐Xin Wu, Hai‐Yan Xu, Shu‐Guang Jian, Xun Gong, Xiu‐Yan Feng

**Affiliations:** ^1^ Key Laboratory for Plant Diversity and Biogeography of East Asia, Kunming Institute of Botany Chinese Academy of Sciences Kunming China; ^2^ Key Laboratory of Economic Plants and Biotechnology, Kunming Institute of Botany Chinese Academy of Sciences Kunming China; ^3^ University of Chinese Academy of Science Beijing China; ^4^ Plant Science Institute, School of Life Sciences Yunnan University Kunming China; ^5^ CAS Engineering Laboratory for Vegetation Ecosystem Restoration on Islands and Coastal Zones, South China Botanical Garden Chinese Academy of Sciences Guangzhou China

**Keywords:** conservation genetics, conservation strategies, *Cycas taiwaniana*, demographic history, genetic diversity and structure

## Abstract

Hainan Island had experienced several cold–warm and dry–humid fluctuations since the Late Pleistocene period, resulting in separating and connecting from the mainland several times with the cyclic rise and fall of sea level. The fluctuations can change the biota and ecological environment in the island. *Cycas taiwaniana* Carruthers is endemic to Hainan Island and is classified as endangered by the International Union for Conservation of Nature (IUCN). To comprehensively understand the genetic dynamics of *C. taiwaniana*, we sampled 12 wild populations in Hainan Island and one cultivated population in Fujian province, and analyzed the genetic diversity, genetic structure, and demographic history based on the molecular data. Results revealed that *C. taiwaniana* had relatively low genetic diversity and high genetic differentiation. Haplotypes of *C. taiwaniana* diversified during the Pleistocene based on the chloroplast DNA (cpDNA) and the concatenated nuclear DNA (nDNA) data. Genetic cluster analyses based on the microsatellite (SSR) data showed that the 12 wild populations were separated into three clusters which could be three evolutionary significant units (ESUs), indicating three basic units of protection were identified. Moreover, we also confirmed the cultivated population FJ derived from the DLS1‐GSL clade. Demographic inference from different data was discordant, but overall, it uncovered that *C. taiwaniana* had experienced population contraction events twice during the Pleistocene and Holocene, and then expanded recently. Our study elucidated the population genetic characteristics of *C. taiwaniana*, and guided us to develop targeted conservation and management strategies for this endangered species.

## Graphical abstract

*Cycas taiwaniana* had relatively low genetic diversity and high genetic differentiation, three evolutionarily significant units were identified and this species had experienced twice population contraction events and expanded recently. By analyzing the genetic diversity, genetic structure and demographic history of *C. taiwaniana* based on DNA data and microsatellites, specific protection recommendations were formulated for *C. taiwaniana*. This study provides guidelines for the development of conservation strategies for other species which distributed on Hainan islands.

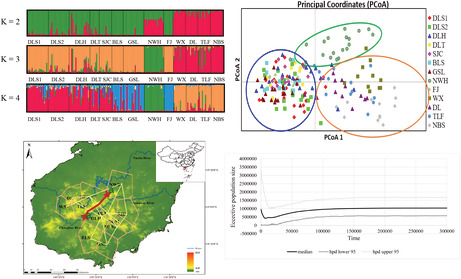

## 1 INTRODUCTION

Conservation genetics plays an important role in the conservation of rare and endangered species. At a time of rapid loss of biodiversity, it is more necessary to study population genetics and historical dynamics of endangered species (Liu & Zhao, 1999; Miao et al., 2021). The application value of population genetics lies in the ability to find the key factors which lead to the loss of rare and endangered species. It provides the maximum and accurate conservation strategy by using DNA data to study the genetic structure and the factors causing the genetic change in populations. Population genetics is becoming more and more popular in animals and plants conservation (Qiao et al., 2021; Sanal Demirci et al., 2021; Shan & Diao, 2011; Wang et al., 2021) and is also widely used in cycads (Feng et al., 2017; Wang et al., 2019; Xiao et al., 2020).

Cycads are one of the oldest and the most ancestral living seed plant which belongs to gymnosperms. They flourished from late Triassic to early Cretaceous of Mesozoic era, and declined gradually until late Cretaceous (Gao & Thomas, 1989). An earlier study showed that living cycad species originated in the past 12 million years (Nagalingum et al., 2011). However, a recent study has shown that cycads are not so young and the mean crown age of extant Cycadaceae is about 69–43 million years (Liu et al., 2021). The newest list of cycads consists of 10 genera and 367 species, and the genus *Cycas* is the largest genus of the extant cycads owning about 118 species (Calonje et al., 2022). According to the latest records, there are about 20 *Cycas* species in China (Xi et al., 2022), which are all listed in The List of First‐level Protected Plants of China. A total of 99 *Cycas* species were included in the IUCN Red List of threatened species and about 81% of *Cycas* species were endangered and needed us to protect them (IUCN, 2022).

*Cycas taiwaniana* was named in 1983 by Willian Carruthers who thought it came from Taiwan based on the words that Hanke had written on the specimen. This species has long been cultivated in China (Guangdong and Fujian Provinces), most of them are from cultivated plants whereas are extremely rare in the wild (Hill, 2008). For a long time, the origin of *C. taiwaniana* has been unknown, and it is generally treated as a *C. taiwaniana* complex. The *C. taiwaniana* complex consisted of *C. fairylakea*, *C. changjiangensis*, *C. hainanensis*, *C. lingshuigensis*, *C. szechuanensis* and *C. taiwaniana*, and they are extremely similar in morphology. Fortunately, a new study treated *C. changjiangensis*, *C. hainanensis*, and *C. lingshuigensis* as synonyms for *C. taiwaniana* by using molecular data and preliminary morphological inference in species delimitation analysis, and verified the origin of *C. taiwaniana* is Hainan Island (Feng et al., 2021).

Hainan Island is considered the second largest island in China. Due to its unique tectonic position which is on the northern edge of the tropics, tropical monsoon climate and rich biological species, it is recognized as a hotspot of biodiversity conservation in the world (Cai et al., 2021). The flora of Hainan Island is dominated by tropical families, genera and species. From the perspective of biogeography, it is proposed that Hainan Island was connected to Vietnam and the Guangxi province of China during the Eocene, and then moved and rotated to the southeast and finally reached the present position (Zhu, 2020). Due to the low latitude of Hainan Island, some ancient plants and animals can be preserved. Some researchers found Cycadaceae palynological fossils of the late Pleistocene strata in Hainan Island (Yan, 2006). Moreover, a biogeographical analysis also showed that the extant *Cycas* species were of South China origin (Xiao & Moller, 2015). Since the Quaternary, Hainan Island may have been connected to Guangdong Leizhou Peninsula several times due to the rise and fall of sea level, which lead to the biota complicated in Hainan Island (Zhu, 2020). *Cycas taiwaniana* was endemic to Hainan Island and distributed mainly in Limuling Mountain and Wuzhi Mountain.

In this study, we used four chloroplast DNA (cpDNA) intergenic spacers, four nuclear genes and 10 microsatellites to perform comprehensive population genetics analysis of *C. taiwaniana*. We aim to: (i) evaluate the genetic diversity, genetic differentiation and genetic clusters of *C. taiwaniana*, (ii) determine the phylogenetic divergence time estimation, (iii) explore the demographic history of *C. taiwaniana* by combining with the geological history of Hainan Island, and (iv) provide the basic and practical guidelines for its conservation strategies.

## 2 MATERIAL AND METHODS

### 2.1 Plant materials

**FIGURE 1:**
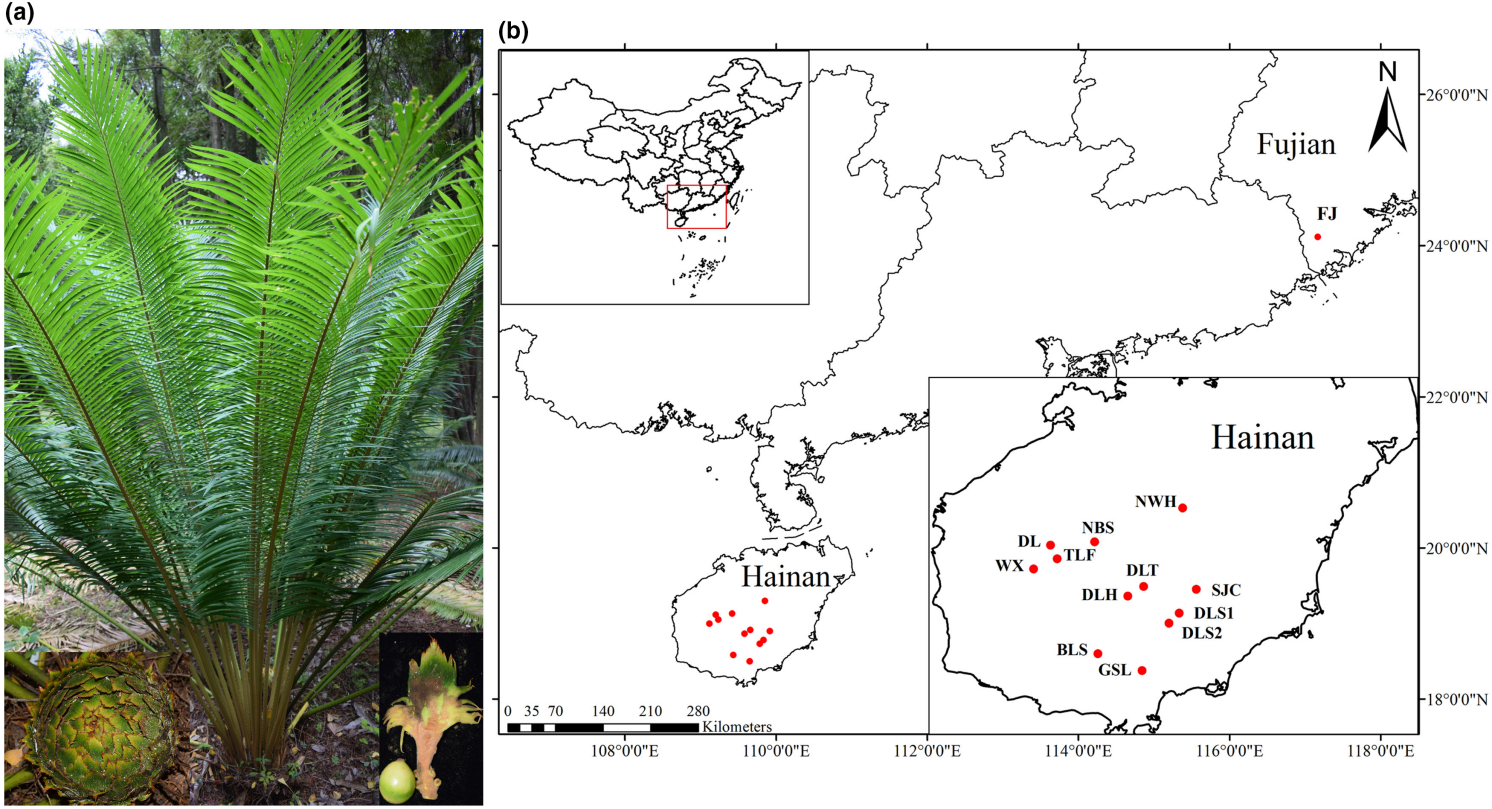
The morphological characteristics and sampling detail distribution of *Cycas taiwaniana*

A total of 13 populations of *C. taiwaniana* were collected by field investigation. Among them, the population FJ was a cultivated population. After collection, fresh and healthy leaves were immediately dried in silica gel. Ten individuals from each population were randomly selected for DNA sequencing, 20 individuals were selected for microsatellite analysis, and the populations with fewer than 10 or 20 individuals were all sampled. In total, we used 128 and 188 individuals for DNA sequencing and microsatellite genotyping, respectively. Sampling detail information and distribution were summarized in Table S1 and Figure 1. Distribution maps were drawn using the ArcGIS v.10.2 (http://desktop.arcgis.com).

### 2.2 Genomic DNA extraction and PCR amplification

Genomic DNA was extracted using the modified CTAB method (Doyle, 1991). We chose four cpDNA intergenic spacers (*atp*B‐*rbc*L, *psb*A‐*trn*H, *psb*M‐*trn*D, and *trn*S‐*trn*G), four nuclear genes (*AC*5, *PHYP*, *PPRC*, and *AAT*) and 10 microsatellites for population genetics analysis, and the details of primers were provided in Table S2. Polymerase chain reaction (PCR) amplification procedures and PCR amplification system were the same as those used in Feng et al. (2014) and Feng, Liu, and Gong (2016). DNA sequencing was carried out by using an ABI 3770 automated sequencer, and PCR products of the targeted microsatellite loci were separated and visualized using an ABI 3730XL automated sequencer at Kunming Branch of Tsingke Biotechnology Co., Ltd. Microsatellite profiles were read with GeneMapper v.4.0 software (Applied Biosystems). All sequences were deposited in GenBank with the accession numbers (Table S3).

### 2.3 Data analysis

#### 2.3.1 Data analysis of DNA sequences

DNA sequences were edited and assembled using the SeqMan (Swindell & Plasterer, 1997). Multiple alignments of DNA sequences were performed in Bioedit v7.0.4.1 (Hall, 1999). We combined the four cpDNA fragments and used the combined cpDNA sequence in the subsequent analyses.

The number of haplotypes, nucleotide diversity (*P*i) and haplotype diversity (*H*d) for 13 populations of *C. taiwaniana* were calculated from aligned DNA sequences in DnaSP v5.0 (Librado & Rozas, 2009). Two measures of population differentiation (*G*_ST_ and *N*_ST_) were calculated in Permut v1.0 (http://www.pierroton.inra.fr/genetics/labo/Software/Permut). Genetic variation assigned within and among populations was performed with an analysis of molecular variance (AMOVA) using the software Arlequin v3.11 (Excoffier et al., 2005). The Network v10.2.0.0 (Bandelt et al., 1999) was applied to estimate the degree of relatedness among cpDNA and nDNA haplotypes with indels treated as single mutational events.

We applied the secondary calibration point (27.9193 Mya) to estimate the divergence time of haplotypes for cpDNA and the concatenated nDNA with *C. szechuanensis* as the outgroup (Liu et al., 2021). Nucleotide substitution models were tested in PhyloSuite v1.2.2 (Lanfear et al., 2017; Zhang et al., 2020). The divergence times were estimated in BEAST v1.6.1 (Drummond & Rambaut, 2007). The HKY with a relaxed lognormal molecular clock model and the GTR + G4 with a relaxed lognormal molecular clock model were used for cpDNA and nDNA, respectively. A normal prior with a mean value at 27.9193 and a stdev value at 0.5 for the Yule birth rate were both used in cpDNA and nDNA. The mutation rate posterior estimates and the time of divergence were obtained by Markov Chain Monte Carlo (MCMC) analysis. Log parameters were sampled every 10,000 iterations under 100,000,000 generations. The analysis ran three times under the same condition and all parameters were stabilized. All the effective sample size parameters were required to exceed 200 checked in TRACER v1.5 (Rambaut & Drummond, 2009). The log and tree files were combined with LogCombiner v1.6.1 and the consensus tree was generated with TreeAnnotator v1.6.1 with the first 25% burn‐in. All the results were checked in the Figtree v.1.4.3 (Rambaut et al., 2016).

To gain more comprehensive inferring and understanding of the historical demography of *C. taiwaniana*, we analyzed the Bayesian Skyline Plot (BSP) of cpDNA data and the four nuclear genes respectively and the Extended Bayesian Skyline Plot (EBSP) of all genes with unlinked model in the BEAST program (Heled & Drummond, 2008). We also used the DnaSP v5.0 software to investigate population dynamics of the species by performing a pairwise mismatch distribution analysis and neutrality tests which included Tajima's *D*, Fu and Li′s D* and F* and Fu's *F*_*S*_ (Fu, 1997). The sum‐of‐squared deviations (SSD) and raggedness index as well as their *p* values were calculated in Arlequin v3.11.

#### 2.3.2 Data analysis of microsatellites

The genetic diversity indices (the number of alleles (*N*_A_), private alleles (*A*_P_), effective number of alleles (*A*_E_), expected heterozygosity (*H*_E_), observed heterozygosity (*H*_O_), Shannon's diversity index (*I*), fixation index (*F*), and percentage of polymorphic loci (PPB)) were calculated in GenAlEx 6.51b2 (Peakall & Smouse, 2012). Allelic richness (*A*_R_), total genetic diversity for species (*H*_T_), and coefficient of gene differentiation (*G*_ST_) were estimated in FSTAT v1.2 (Goudet, 1995). Using the software Arlequin v3.11 to conduct AMOVA analysis. The pairwise differentiation coefficient (*F*_ST_) between populations (1000 permutations) were calculated in Arlequin v3.11. Gene flows between pairs of populations were tested based on Wright's principles *Nm* = (1 − *F*_ST_)/4*F*_ST_.

A Bayesian analysis of population structure was conducted with STRUCTURE v2.3.4 (Pritchard et al., 2000). The analysis was conducted under the admixture model and correlated allele frequencies, with the number of genetic clusters (K) set to (K = 1–20). Each K was run with 20 independent iterations and each run included a burn in of 100,000 iterations and 100,000 subsequent MCMC steps. The best‐fit number of groupings was evaluated using Delta K in STRUCTURE HARVESTER v0.6.8 (Earl & VonHoldt, 2012). Clumpp v1.1.2 (Jakobsson & Rosenberg, 2007) was used for data integration, and the software Distruct v1.1 (Rosenberg, 2004) was used to visualize the integration results. Based on genetic distances, an individual‐based principal coordinate analysis (PCoA) was visualized in GenAlEx 6.51b2.

The effective population sizes (*N*e) of each population were tested under random mating system in LDNe (Waples & Do, 2008). To ensure the credibility of the analysis results, we chose the lowest allele frequency at 0.05 level with a 95% confidence interval. We detected the bottleneck effect at the population level to explore population demography using two methods (Sign tests and Wilcoxon tests) under the two models: stepwise mutation model (SMM) and two‐phased model (TPM). A mode shift model was also used to test for bottlenecks in each population in BOTTLENECK v1.2.02 (Cornuet & Luikart, 1996; Piry et al., 1999). Parameters for TPM were set up as Variance = 30.00, Probability = 90.00% and estimation based on 10,000 replications. We further investigated a genetic bottleneck using the Garza–Williamson index (Garza & Williamson, 2001) which was calculated in Arlequin v3.11.

## 3 RESULTS

### 3.1 Genetic variations

Total length of the combined cpDNA was 3525 bp (*atp*B‐*rbc*L: 713 bp; *psb*A‐*trn*H: 591 bp; *psb*M‐*trn*D: 1290 bp; *trn*S‐*trn*G: 931 bp), and 11 haplotypes (taiH1‐taiH11) were identified (Table S4). The nucleotide diversity (*Pi*) and the total haplotype diversity (*H*d) of *C. taiwaniana* were 0.00035 and 0.730, respectively (Table 1). Among the 13 populations, only the population GSL had haplotype diversity (*H*d = 0.200) and nucleotide diversity (*P*i = 0.0004). The remaining 12 populations did not have the nucleotide diversity and haplotype diversity (*H*d = 0, *P*i = 0).

**TABLE 1:** Haplotype diversity (*H*d) and nucleotide diversity (*P*i) estimated from cpDNA and nuclear genes

Population code	cpDNA		*AC*5		PHYP		PPRC		AAT	
	*H*d	*P*i × 10^3^	*H*d	*P*i × 10^3^	*H*d	*P*i × 10^3^	*H*d	*P*i × 10^3^	*H*d	*P*i × 10^3^
DLS1	0	0	0.668	1.68	0.511	0.58	0.468	1.33	0.879	7.49
DLS2	0	0	0.758	1.73	0.542	0.62	0.542	1.28	0.800	6.40
DLH	0	0	0.616	0.76	0.758	1.42	0.511	1.47	0.816	7.06
DLT	0	0	0.758	1.60	0.660	1.85	0.503	0.71	0.641	5.24
SJC	0	0	0.353	1.74	0.416	0.47	0.732	5.17	0.805	7.40
BLS	0	0	0.700	1.48	0.395	0.42	0.268	0.38	0.747	5.09
GSL	0.200	0.40	0.747	1.82	0.521	0.56	0.442	0.62	0.805	6.05
NWH	0	0	0.521	0.56	0.795	2.72	0.521	0.73	0.884	7.84
FJ	0	0	0	0	0.529	2.27	0.529	2.24	0.529	4.92
WX	0	0	0.826	3.19	0.705	2.10	0.568	2.22	0.816	7.07
DL	0	0	0.500	0.98	0.779	2.87	0.500	1.12	0.953	11.71
TLF	0	0	0.658	1.51	0.642	1.58	0.484	0.89	0.889	8.89
NBS	0	0	0.642	1.68	0.789	3.32	0.484	0.74	0.800	7.27
Total	0.730	0.35	0.756	1.87	0.735	2.17	0.617	1.88	0.896	8.64

The alignment length of *AC*5 was 923 bp with 15 polymorphic sites, detecting 17 haplotypes (taiA1‐taiA17) in 128 individuals of 13 populations (Table S4). The *P*i and the *H*d of *C. taiwaniana* based on the nuclear gene *AC*5 were 0.00187 and 0.756, respectively (Table 1). The alignment length of *PHYP* was 932 bp with 11 polymorphic sites, detecting 12 haplotypes (taiP1‐taiP12). The *P*i and the *H*d of *C. taiwaniana* were 0.00217 and 0.735, respectively (Table 1). The alignment length of *PPRC* was 710 bp with 19 polymorphic sites, and 11 haplotypes (taiR1‐taiR11) were identified. The *P*i and the *Hd* of *C. taiwaniana* based on the nuclear gene *PPRC* were 0.00188 and 0.617, respectively (Table 1). The alignment length of *AAT* was 538 bp with 29 polymorphic sites, detecting 43 haplotypes (taiT1‐taiT43). The *P*i and the *H*d of *C. taiwaniana* based on the nuclear gene *AAT* were 0.00864 and 0.896, respectively (Table 1).

The genetic diversity of different populations revealed by different DNA sequence data was inconsistent. Except the population GSL, the other 12 populations had no genetic diversity based on cpDNA data (*H*d = 0, *P*i = 0). Four nuclear genes showed variable genetic diversity within 13 populations. For gene *AC*5, population FJ had no genetic diversity (*H*d = 0, *P*i = 0) and population WX had the highest haplotype diversity (*H*d = 0.826, *P*i = 0.00319). For gene *PHYP*, population BLS had the lowest genetic diversity (*H*d = 0.395, *P*i = 0.00042) and population NWH had the highest haplotype diversity (*H*d = 0.795, *P*i = 0.00272). For gene *PPRC*, population BLS had the lowest genetic diversity (*H*d = 0.268, *P*i = 0.00038) and population SJC had the highest haplotype diversity (*H*d = 0.732, *P*i = 0.00517). For gene *AAT*, population DLT had the lowest genetic diversity (*H*d = 0.641, *P*i = 0.00524) and population WX had the highest haplotype diversity (*H*d = 0.953, *P*i = 0.01171).

A total of 120 alleles were identified at the 10 microsatellite loci (Table S5). Diversity estimates varied in different populations (Table 2). There was no private allele in populations DLS2, BLS and NWH, but the population DL had the most private alleles (*N*_P_ = 5). Allelic richness was lowest in population FJ (*A*_R_ = 2.200) and highest in population DL (*A*_R_ = 5.171). The number of alleles (*N*_A_) ranged from 2.200 to 6.600, and the effective number of alleles (*N*_E_) ranged from 1.889 to 3.756. Shannon's diversity index (*I*) ranged from 0.587 to 1.356, and population FJ had the lowest *I* value but population DL had the highest *I* value. Observed heterozygosity (*H*_O_) ranged from 0.375 to 0.648 and expected heterozygosity (*H*_E_) ranged from 0.376 to 0.648, respectively. The population DL had the highest *H*_E_ and population FJ had the lowest *H*_E_. The percentages of polymorphic loci (*PPB*) were high, ranging from 70% to 100%. To sum up, the population FJ had the lowest genetic diversity while the population DL had the highest genetic diversity.

**TABLE 2:** Genetic diversity within populations of *Cycas taiwaniana* based on the microsatellites data

Population	*N*_P_	*A*_R_	*N*_A_	*N*_E_	*I*	*H*_O_	*H*_E_	*F*	PPB (%)	*N*e
DLS1	1	4.963	6.500	3.450	1.268	0.434	0.576	0.216	90.00	438.2 (41.9‐∞)
DLS2	0	4.877	6.600	3.303	1.238	0.420	0.569	0.230	90.00	431.1 (42.5‐∞)
DLH	1	4.953	6.500	3.184	1.318	0.455	0.621	0.374	100.00	61.9 (24.8‐∞)
DLT	1	4.400	4.400	2.887	1.072	0.478	0.543	0.120	90.00	—
SJC	1	4.330	4.900	3.025	1.138	0.542	0.567	0.092	90.00	63.6 (12.2‐∞)
BLS	0	4.289	4.600	2.813	1.062	0.464	0.533	0.122	90.00	18.5 (4.8‐∞)
GSL	1	4.129	5.100	2.844	1.072	0.400	0.518	0.196	90.00	235.0 (33.5‐∞)
NWH	0	4.267	5.000	3.139	1.145	0.479	0.562	0.133	90.00	38.2 (17.8–339.6)
FJ	2	2.200	2.200	1.889	0.587	0.578	0.376	−0.613	70.00	—
WX	2	3.538	3.800	2.378	0.883	0.383	0.457	0.170	90.00	4.5 (2.1–14.7)
DL	5	5.171	5.600	3.756	1.356	0.567	0.648	0.131	90.00	10.8 (4.9–29.1)
TLF	2	4.228	4.500	3.056	1.176	0.475	0.613	0.195	100.00	—
NBS	3	3.610	3.900	2.510	0.906	0.375	0.468	0.242	100.00	13.1 (3.3–1187.0)
Mean	1.31	4.235	4.892	2.941	1.094	0.465	0.542	0.140	90.77	1314.9

*Note*: The 95% confidence intervals are in parentheses.

Abbreviations: —, null data; *A*_E_, the effective number of alleles; *A*_R_, allelic richness; *F*, fixation index; *H*_E_, expected heterozygosity; *H*_O_, observed heterozygosity; *I*, Shannon's information index; *N*_A_, number of alleles; *N*e, effective population size; *N*_P_, private alleles; *PPB*, the percentage of polymorphic loci.

The AMOVA results showed that there were more variations partitioned among populations (98.5%) than within populations (1.5%) based on the cpDNA data, whereas there were fewer variations partitioned among populations (23.59%, 28.33%, 24.05%, and18.49%) than within populations (76.41%, 71.67%, 75.95%, and 81.51%) based on the four nuclear genes (*AC*5, *PHYP*, *PPRC*, and *AAT*), respectively. Moreover, more variations were partitioned within populations (81.74%) than among populations (18.26%) based on the SSR data. The *F*_ST_ value for the three types of markers ranged from 0.183 to 0.985 with significance, indicating highly significant genetic differentiation among the 13 populations of *C. taiwaniana* (Table 3). According to the formula (*N*m = (1 − *F*_ST_)/4*F*_ST_), gene flow was inversely proportional to the *F*_ST_ value. The genetic differentiation between populations based on the cpDNA data was significant. Therefore, there was almost no gene flow between populations. Whereas the genetic differentiation between populations was smaller and the gene flow between populations was larger based on nuclear data. Gene flows between each pair of the 13 populations based on the SSR data were shown in Table S6. Population DLS2 had the most gene flow with the population DLS1 (*Nm* = 8.452), and the population NBS had the least with the population FJ (*Nm* = 0.330). Fixation indices (*F*) were positive for all 13 populations, with a mean value *F* = 0.140, which suggests a high level of inbreeding within each population (Table 2).

**TABLE 3:** Analysis of molecular variance (AMOVA) based on DNA sequences and microsatellites for populations of *Cycas taiwaniana*

Marker	Source of variation	df	Sum of squares	Variance components	Percentage of variation (%)	*F*_ST_	*N*m
cpDNA	Among populations	12	1181.055	9.98157	98.50	0.985***	0.00381
	Within populations	115	17.500	0.15217	1.50		
*AC5*	Among populations	12	56.942	0.20695	23.59	0.236***	0.80932
	Within populations	243	162.906	0.67039	76.41		
*PHYP*	Among populations	12	78.138	0.29304	28.33	0.283***	0.63339
	Within populations	243	180.167	0.74143	71.67		
*PPRC*	Among populations	12	44.770	0.16328	24.05	0.240***	0.79167
	Within populations	243	125.328	0.51575	75.95		
*AAT*	Among populations	12	126.047	0.43588	18.49	0.185***	1.10135
	Within populations	243	466.894	1.92138	81.51		
SSR	Among populations	12	253.350	0.63628	18.26	0.183***	1.11612
	Within populations	363	1033.703	2.84767	81.74		

*** *p* < .001.

*N*_ST_ was significantly greater than *G*_ST_ (*p* < .05) based on the nuclear gene *ATT* and *PHYP*, indicating that *C. taiwaniana* had significant phylogeographical structure (Table S7). However, the rest two nuclear genes and cpDNA showed *C. taiwaniana* had no distinct phylogeographical structure.

### 3.2 Network of haplotypes and divergence time estimation

**FIGURE 2:**
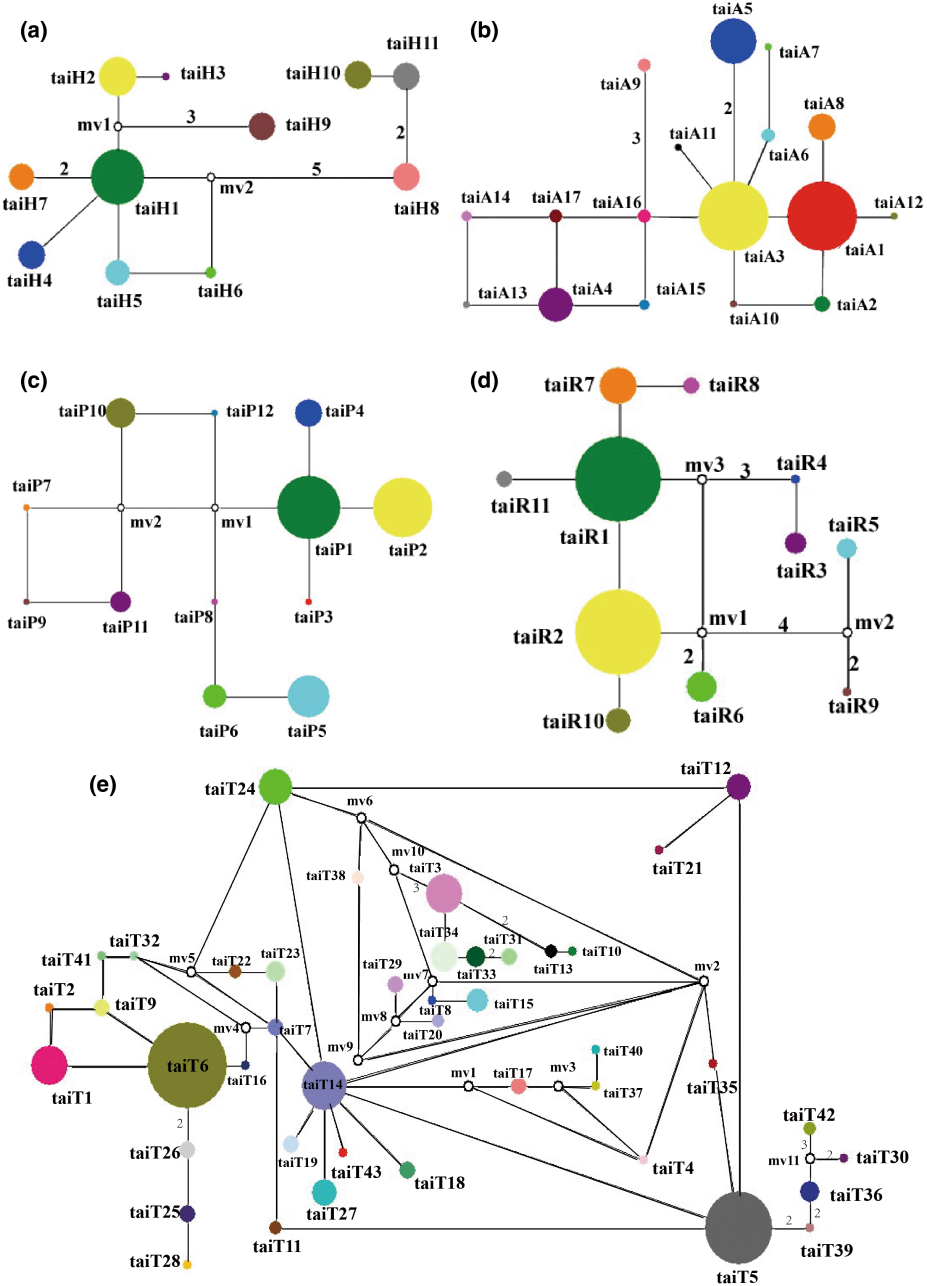
Network of haplotypes of *Cycas taiwaniana* based on cpDNA (a), AC5 (b), PHYP (c), PPRC (d), and AAT (e). The numbers on branches indicate mutational steps. Haplotype distribution in 13 populations refers to Table S4.

**FIGURE 3:**
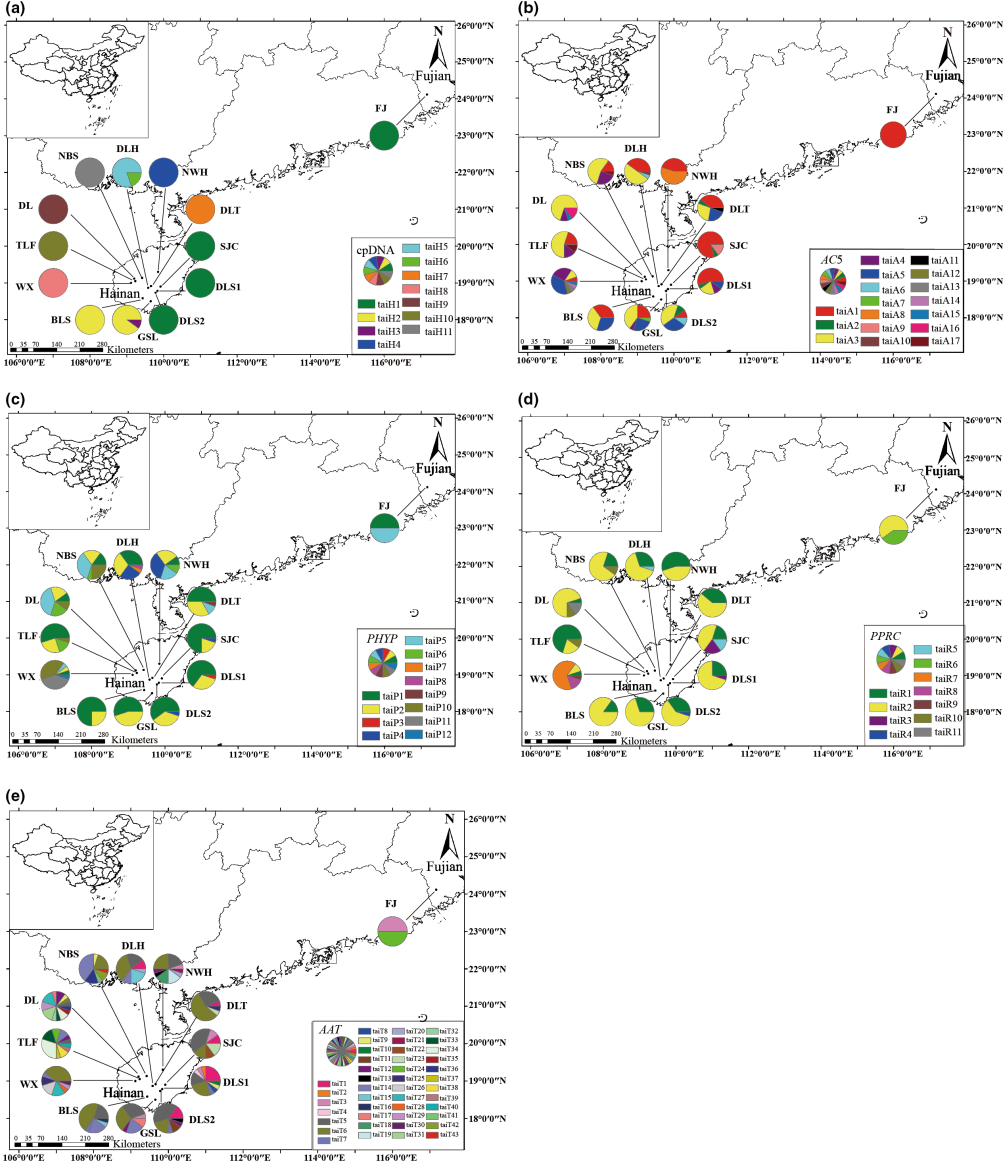
Geographical distribution of 13 populations of *Cycas taiwaniana* and distribution of its haplotypes detected from cpDNA (a), AC5 (b), PHYP (c), PPRC (d), and AAT (e). Population codes refer to Table S1.

Genealogies reflecting haplotypes topology and frequency were constructed based on cpDNA and four nuclear genes (*AC5*, *PHYP*, *PPRC*, and *AAT*; Figures 2 and 3). In the cpDNA haplotype network, the haplotype taiH1 was located at the internal node and appeared the most frequently. Based on the nuclear genes data, the highest frequency haplotype was always distributed in each population. For the nuclear gene *AC5*, the highest frequency haplotypes were taiA1 and taiA3, and for the nuclear gene *PHYP*, the highest frequency haplotypes were taiP1 and taiP2. TaiR1 and taiR2 were the highest frequency haplotypes in *C. taiwaniana* based on the nuclear gene *PPRC* and taiT5 and taiT6 were the highest frequency haplotypes based on the nuclear gene *AAT*. All the networks had closed loops which may be due to the recent reverse or parallel mutation of those genes, or possible recombination events in those genes. In the recombination test, the four nuclear genes all had recombination events, the minimum numbers of recombination events in *AC*5, *PHYP*, *PPRC*, and *AAT* were three, two, one, and eight, respectively.

**FIGURE 4:**
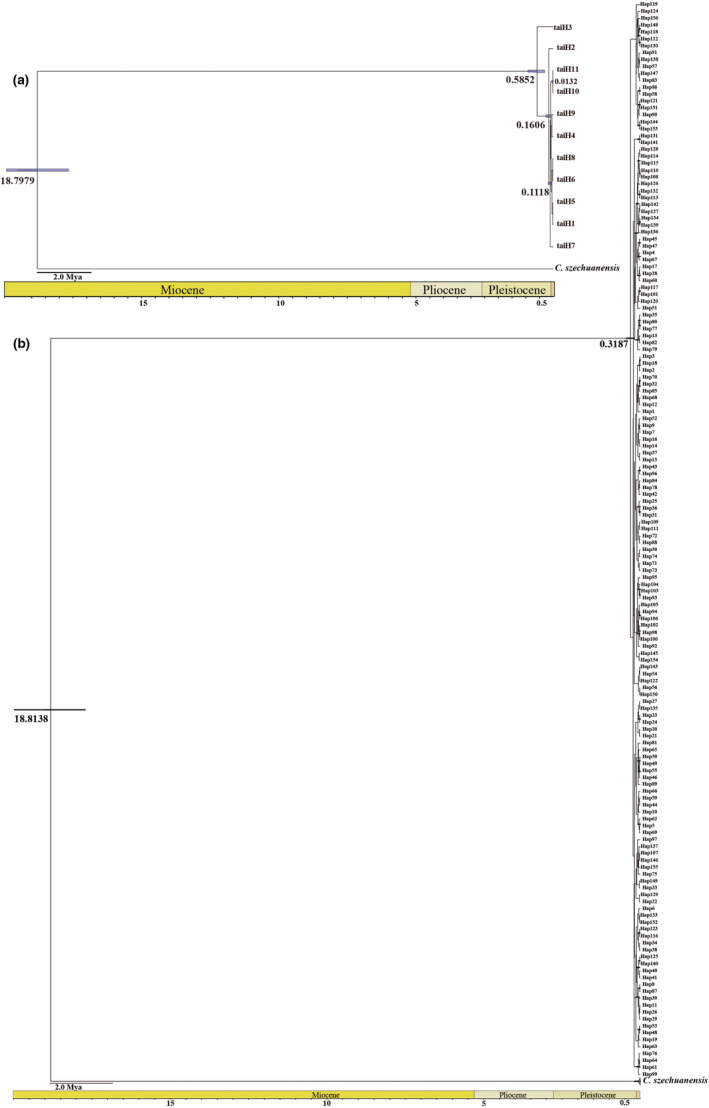
The BEAST‐derived tree based on cpDNA (a) and the concatenated nDNA (b) haplotypes. The numbers on nodes represent divergence time (Mya) and the bars indicate the 95% highest posterior densities.

According to the secondary calibration point, we inferred the divergence time between *C. taiwaniana* and the outgroup *C. szechuanensis* were 18.7979 Mya and 18.8138 Mya based on cpDNA and nDNA, respectively. The divergence time in the crown node of haplotypes were 0.5852 Mya (cpDNA) and 0.3187 Mya (nDNA), which indicates that haplotypes diverged in the Pleistocene (Figure 4).

### 3.3 Genetic cluster and Barrier analysis

**FIGURE 5:**
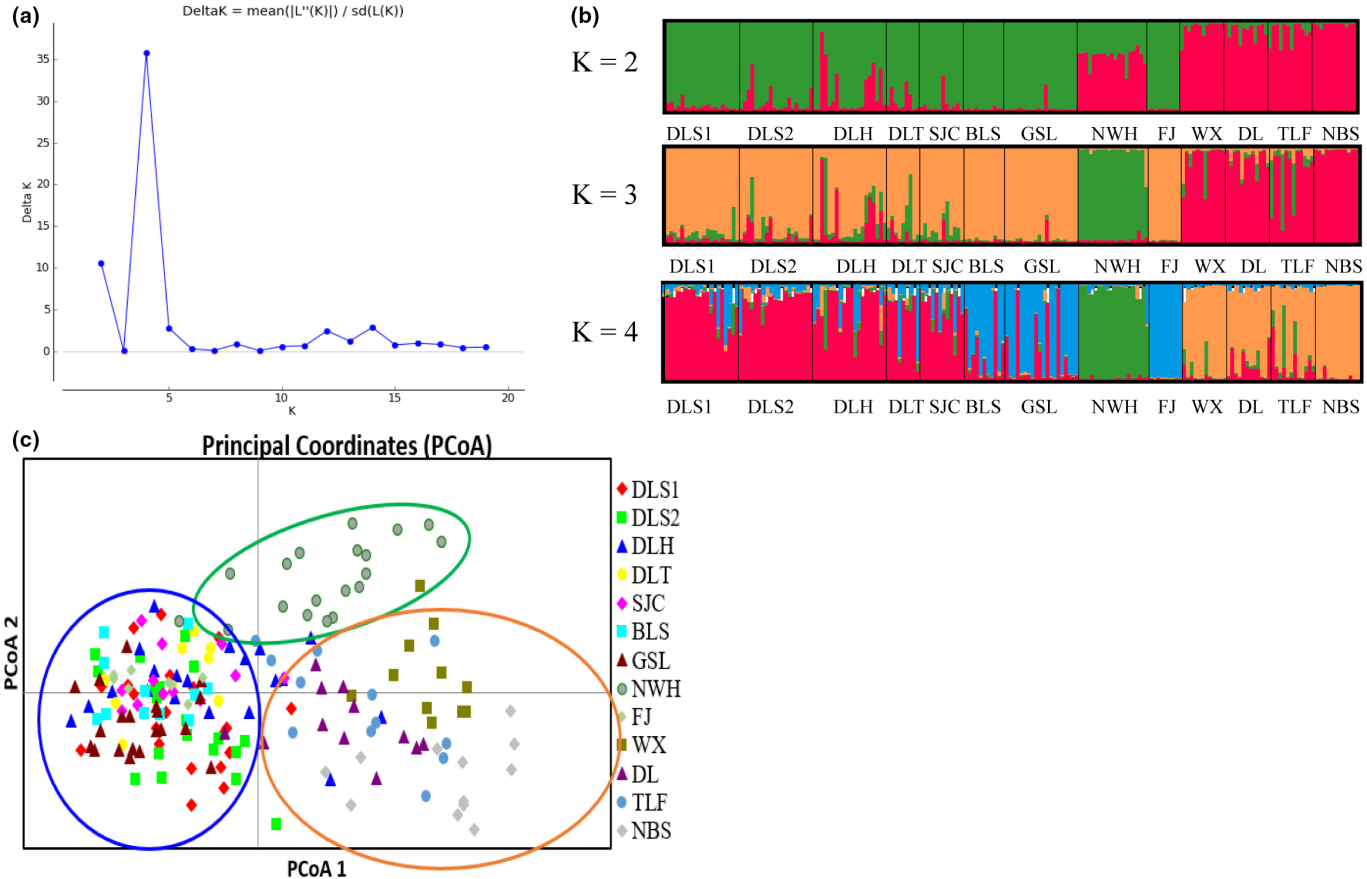
(a, b) Bayesian inference using STRUCTURE of *Cycas taiwaniana*; (c) Principal coordinate analysis of SSR data from 13 populations of 188 individuals of *C. taiwaniana*.

The STRUCTURE analysis showed that the optimal K value was K = 4 (Figure 5a,b), which showed that the 13 populations were clustered into four groups. Populations DLS1, DLS2, DLH, DLT, SJC, BLS, and GSL were grouped into one cluster (Cluster I), population NWH was grouped into the second cluster (Cluster II), WX, DL, TLF, and NBS were grouped into a third cluster (Cluster III), population FJ was grouped into the fourth cluster (Cluster IV). However, when the K value was 3, population FJ was grouped into the cluster I. The result of the PCoA analysis (Figure 5c) showed that two‐dimensional PCoA separated all individuals into three clusters along the two axes. The first group consisted of populations DLS1, DLS2, DLH, DLT, SJC, BLS, GSL, and FJ, the second group only had a population NWH, and the third group owned population WX, DL, TLF and NBS.

**FIGURE 6:**
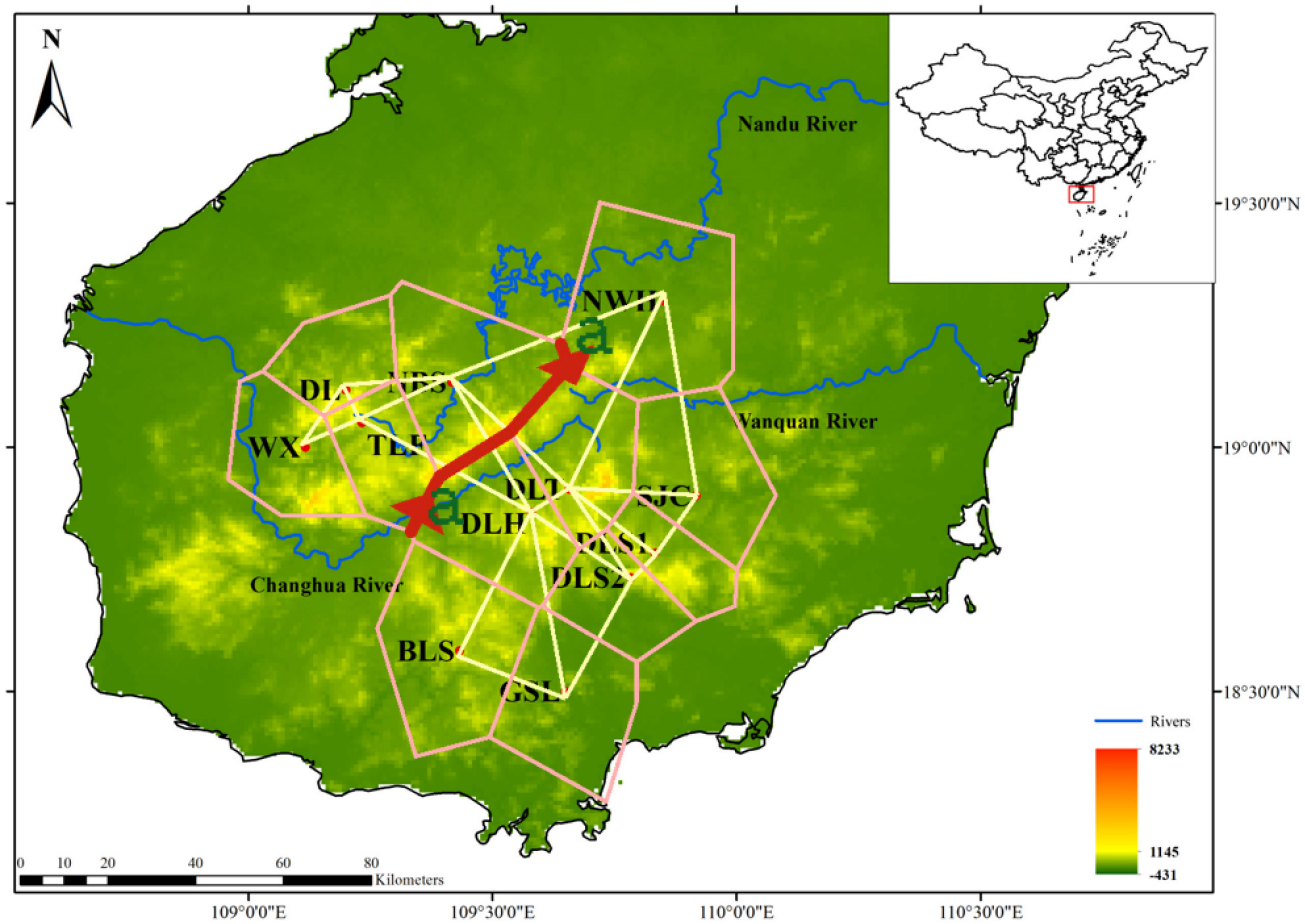
The boundaries detected using the Barrier program based on matrices of Nei and Chesser (1983) unbiased genetic distance.

The Barrier analysis showed that there was only one major genetic boundary (Barrier I), with a 50.8% mean support value, separating the 12 populations into two clusters (Figure 6), the first group had population WX, DL, TLF, and NBS, the second group had the remaining eight populations of *C. taiwaniana*. According to the map verification, the isolation of two groups of *C. taiwaniana* might be caused by the obstruction of Changhua River.

### 3.4 Population history dynamics

**FIGURE 7:**
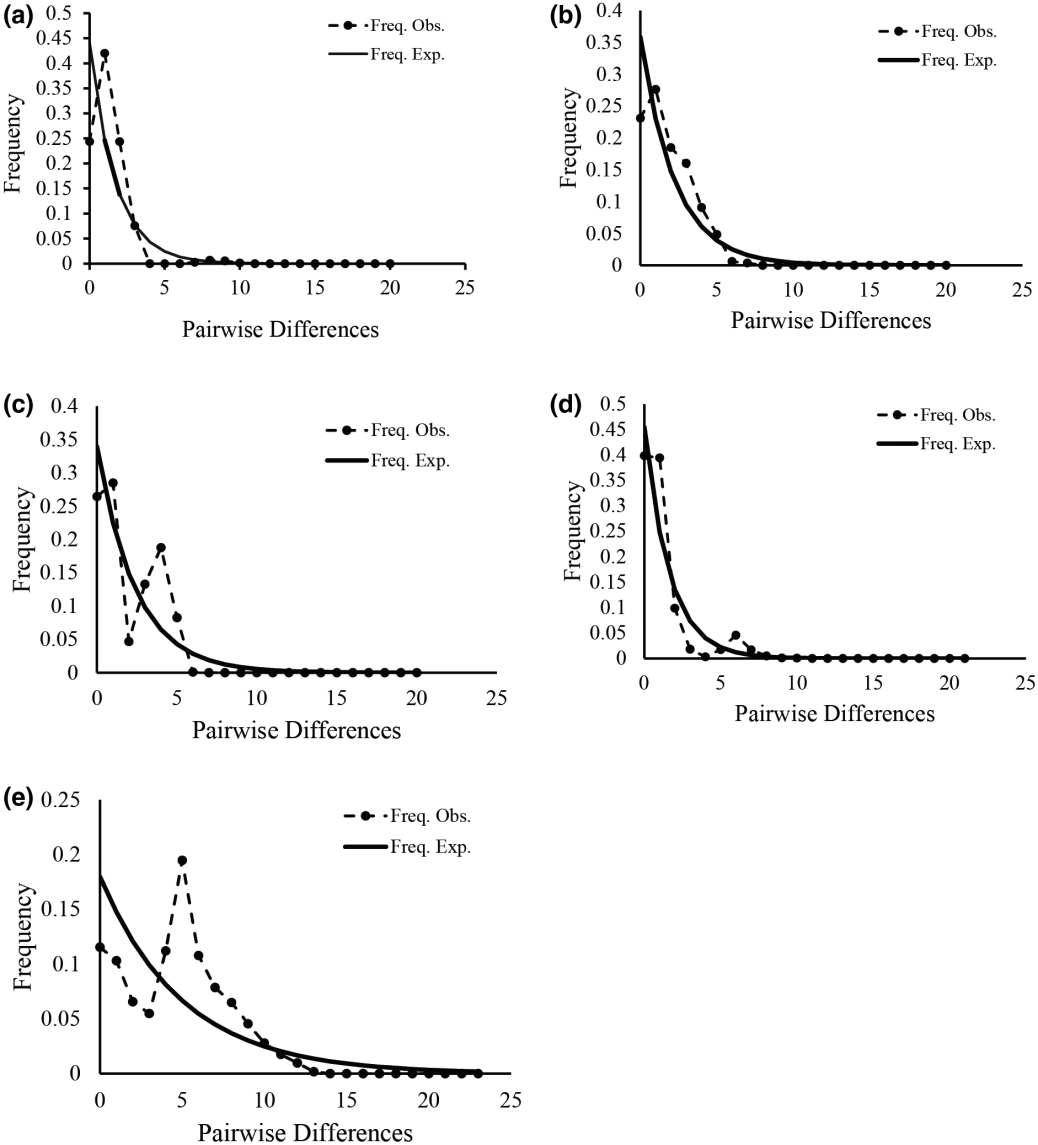
Mismatch distribution of cpDNA (a) and nDNA (b: *AC*5, c: *PHYP*, d: *PPRC*, e: *AAT*) haplotypes based on pairwise sequence difference against the frequency of occurrence for *Cycas taiwaniana*.

Values for neutrality test and mismatch distribution analysis were listed in Table S8 and Figure 7. Fu's *Fs* was more sensitive to population expansion than the other three neutrality test parameters. Based on nuclear genes *AC*5 and *AAT*, Fu's *Fs* were significant negative values, SSD and raggedness index were not significant, and the mismatch distribution analyses showed a unimodal curve, indicating that *C. taiwaniana* experienced an expansion event (Figure 7). Based on cpDNA data, only Fu and Li's D* and F* were significantly negative and SSD value was significant, suggesting that *C. taiwaniana* might have experienced recent expansion. Based on nuclear gene *PHYP* and *PPRC*, all neutrality test parameters were not significant, indicating *C. taiwaniana* was at demographic equilibrium (Figure 7).

**FIGURE 8:**
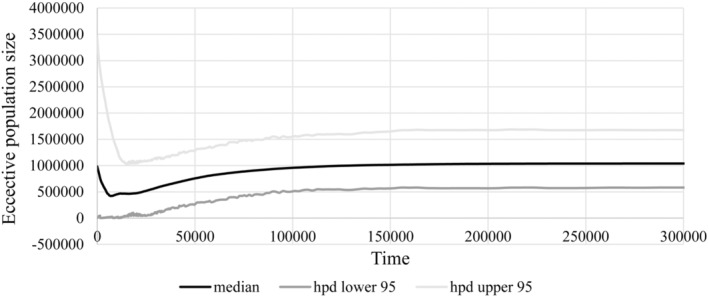
The Extend Bayesian Skyline Plot based on cpDNA and the four nuclear genes for the estimate of fluctuations in effective population size over time. Black line: median estimation; area between gray lines: 95% confidence interval.

Population demographic histories inferred by BSP based on cpDNA data uncovered that *C. taiwaniana* began to experience population constriction at about 100 Kya (Figure S1a). Historical demography deduced from genes *AC*5 and *PPRC* revealed that *C. taiwaniana* had experienced population expansion mainly during the Holocene (Figure S1b,d). However, population demographic histories deduced from gene *PHYP* and *AAT* revealed that *C. taiwaniana* had experienced population constriction during the Pleistocene and then experienced population expansion mainly in the Holocene (Figure S1c,e). EBSP can provide more detailed evidence of demographic history. *C. taiwaniana* had experienced bottleneck effect in history. After experiencing a long time (200–20 Kya) population decline, it continued for a short stable period (20–10 Kya), and then experienced small‐scale population decline. Until 7 Kya, the species began to expand (Figure 8).

The bottleneck analyses were used to calculate heterozygosity excess test and mutation‐drift equilibrium as estimated with different models and different methods (Table 4). Based on the model TPM and SMM, the population DLS1, DLS2, DLH, SJC, BLS, and GSL showed a significant excess of heterozygosity (*p* < .05). Under the Mode shift models, the 12 populations had normal L‐shaped distributions, suggesting that *C. taiwaniana* had not experienced a recent severe bottleneck. Compared with the bottleneck analysis, Garza–Williamson indices can reflect earlier population decline. GWIs of the 12 populations were lower than the critical Mc value of 0.68, which indicating that *C. taiwaniana* had experienced bottleneck effect in history (Table 4).

**TABLE 4:** Bottleneck analysis for 13 populations of *Cycas taiwaniana*

Population	T.P.M		S.M.M		Mode shift	Garza–Williamson Index
	Sign test	Wilcoxon test	Sign test	Wilcoxon test		
DLS1	0.105	0.049*	0.004**	0.006**	L	0.342
DLS2	0.006**	0.010*	0.005**	0.004**	L	0.343
DLH	0.017*	0.010*	0.017*	0.005**	L	0.324
DLT	0.116	0.426	0.120	0.203	L	0.312
SJC	0.023*	0.203	0.027*	0.020*	L	0.281
BLS	0.029*	0.129	0.026*	0.037	L	0.299
GSL	0.034*	0.010*	0.000**	0.002**	L	0.291
NWH	0.282	0.652	0.106	0.301	L	0.324
WX	0.324	0.570	0.324	0.359	L	0.290
DL	0.533	0.734	0.543	1.000	L	0.289
TLF	0.326	0.246	0.434	1.000	L	0.317
NBS	0.467	0.375	0.239	0.160	L	0.312

* *p* < .05, significant difference

** *p <* .01, most significant difference.

## 4 DISCUSSION

### 4.1 Low genetic diversity and genetic differentiation

Genetic diversity, species diversity, and ecosystem diversity are three main pillars of biodiversity. On the basis of principle of population genetics, the conservation of biodiversity is ultimately the conservation of genetic diversity (Liu & Zhao, 1999). The early evaluation on genetic diversity of species mainly used allozymes. Based on allozyme estimation, the levels of genetic diversity of some cycads were similar (*Microcycas calocoma* (*A* = 1.49, *p* = 48.09% and *H*e = 0.17); *Dioon sonorense* (*A* = 2, *p* = 81.6% and *H*e = 0.314); *Zamia loddigesii* (*A* = 1.80, *p* = 66.6% and *H*e = 0.266); *D. edule* (*A* = 1.44, *p* = 54.78% and *H*e = 0.24)) (Gonzalez‐Astorga et al., 2003, 2006; González‐Astorga et al., 2008; Pinares et al., 2009). In this study, we explored the genetic diversity of *C. taiwaniana* by estimating genetic variation parameters from three types of DNA data. By comparing the genetic diversity of *C. taiwaniana* with other cycad species, we got an objective evaluation.

Based on cpDNA data, the genetic diversity of *C. taiwaniana* was generally low, whose *P*i was lower than *C. segmentifida*, *C. chenii*, *C. debaoensis*, *C. bifida*, and *C. diannanensis*, but the *H*d was higher than those of the above five species. The nucleotide diversity and haplotype diversity of *C. taiwaniana* was lower than *C. simplicipinna*, *C. panzhihuaensis*, *C. multipinnata*, *C. dolichophylla*, and *C. guizhouensis* (Table 5; Zheng et al., 2017).

**TABLE 5:** Comparison of genetic diversity of *Cycad* species based on the cpDNA data

Species	*Pi*	*H*d	References
*C. taiwaniana*	0.00035	0.730	This study
*C. segmentifida*	0.00229	0.602	Feng et al. (2017)
*C. chenii*	0.00143	0.621	Yang et al. (2017)
*C. debaoensis*	0.00132	0.49160	Zhan et al. (2011)
*C. bifida*	0.00191	0.7184	Gong (2015)
*C. diannanensis*	0.00087	0.564	Liu et al. (2015)
*C. simplicipinna*	0.00259	0.864	Feng et al. (2014)
*C. panzhihuaensis*	0.00259	0.8033	Xiao et al. (2020)
*C. multipinnata*	0.00149	0.7718	Gong et al. (2015)
*C. dolichophylla*	0.00251	0.940	Zheng et al. (2016)
*C. guizhouensis*	0.00091	0.794	Feng, Zheng, and Gong (2016)

Due to the different evolutionary history of nuclear genes, they reveal inconsistent genetic diversity for *C. taiwaniana*. Based on the nuclear gene *PHYP*, the genetic diversities of *C. taiwaniana* (*H*d = 0.735, *Pi* = 0.00217), *C. chenii* (*H*d = 0.788, *Pi* = 0.0027; Yang et al., 2017), and *C. segmentifida* (*H*d = 0.700, *Pi* = 0.00231; Feng et al., 2017) were similar; the genetic diversity of *C. taiwaniana* was higher than *C. panzhihuaensis* (*H*d = 0.3302, *Pi* = 0.0004; Xiao et al., 2020) and *C. guizhouensis* (*H*d = 0.555, *Pi* = 0.00062; Feng, Zheng, & Gong, 2016).

According to the microsatellites results, the *PPB* value (90.77%) of *C. taiwaniana* was similar to other *Cycas* species (The *PPB* values for *C. multipinnata*, *C. dolichophylla*, *C. guizhouensis*, *C. simplicipinna*, *C. segmentifida*, *C. chenii*, and *C. debaoensis* were 94.12%, 87.98%, 88.11%, 90.63%, 84.52%, 95.92%, and 93.58%, respectively), and higher than *C. panzhihuaensis* (72.23%), *C. megacarpa* (61.4%), *Z. incognita*, and *Z. melanorrhachis* (69.3%–78.9%) (Aristizábal et al., 2017; James et al., 2018); the *H*_E_ and *N*_E_ of *C. taiwaniana* had the similar sizes with those *Cycas* species (Feng et al., 2014, 2017; Feng, Zheng, & Gong, 2016; Gong et al., 2015; James et al., 2018; Xiao et al., 2020; Yang et al., 2017; Zhan et al., 2011; Zheng et al., 2016).

The AMOVA results were consistent with those of other studies, which showed that the genetic differentiations of many *Cycas* species were distributed among populations based on the cpDNA data (Feng et al., 2014, 2017; Feng, Zheng, & Gong, 2016; Wang et al., 2019; Xiao et al., 2020; Yang et al., 2017; Zheng et al., 2016). Moreover, nuclear genes explored the opposite results (Feng et al., 2014; Feng, Zheng, & Gong, 2016; Wang et al., 2019; Xiao et al., 2020; Yang et al., 2017), genetic variations were mainly distributed within populations with the species *C. dolichophylla* as an exception, whose genetic variations among populations was greater than that within populations (Zheng et al., 2016). In *Cycas*, the chloroplast DNAs are maternal inheritance (Zhong et al., 2011), and genetic materials are transmitted by seeds. While, the nuclear genes are biparental inheritance, and genetic materials are transmitted by both seeds and pollens. The seeds of *Cycas* often fall near the mother plant, resulting in increased inbreeding, which increased homogenization within the population and heterogeneity among populations. Moreover, the evolution rate of nuclear genes was faster than that of chloroplast and mitochondrial, nuclear genes can accumulate more variations. Therefore, cpDNA data revealed that more genetic variations were distributed among populations.

### 4.2 Significant genetic structure and the origin of cultivated population

Previous studies had shown that there were low levels of gene flow among populations in most cycads, however, the gene flow among some populations of *C. taiwaniana* was larger in our study (Aristizábal et al., 2017). The seeds of *Cycas* species were so heavy that they could only be disperse closely by rodents or gravity. One study had shown that the gene flow of *Cycas* was 2–7 km (Yang & Meerow, 1996), leading to introgression and enhancing the probability of inbreeding (Hall & Walter, 2011). According to the biological significance, we considered it more reasonable to divide the 13 populations of *C. taiwaniana* into three clusters, and the cultivated population FJ was clustered into the DLS1‐GSL clade. In terms of genetic components, we inferred that the population FJ probably came from the DLS1‐GSL clade (Figure 5). The genetic cluster results (STRUCTURE analysis and PCoA) all showed that the populations WX, DL, TLF, and NBS had significant genetic differentiation from the other eight wild populations. Weak gene flow between these four populations and the other eight wild populations of *C. taiwaniana* was revealed (Table S6). Hainan island is high in the middle and low in the periphery, with Wuzhishan and Yinggeling as the uplift core (Zuo et al., 2021). Populations WX, DL, TLF, and NBS of *C. taiwaniana* were mainly distributed around Yingge Mountain at 1811 m altitude, while the rest eight wild populations were mostly distributed in Wuzhi Mountain, the two mountains are separated by Changhua River which is the second longest river in Hainan Island. This might be one of the reasons why the four populations genetically were separated from other populations.

### 4.3 Geological history and fluctuating of population historical dynamics

Climate fluctuation during the Quaternary period had effects on the population historical dynamics of *Cycas* species, and different species had different responses to glacial and interglacial influences (Wang et al., 2019). The mismatch distribution analysis of nuclear genes *AC*5, *PPRC*, and *AAT* showed a unimodal curve, which were consistent with the BSP results, indicating that *C. taiwaniana* experienced an expansion event. Other genes (cpDNA and *PHYP*) were inconsistent with the BSP results. When the general population experienced expansion or continuous growth in the past, the mismatch distribution curve showed a unimodal Poisson distribution, and the neutrality test significantly deviated from neutral mutation. However, when the population size remained stable, the mismatch distribution analysis showed a multimodal curve distribution, and the neutrality test was not significant. Actually, demographic histories inferred are more complex than the parametric models involved in these approaches. The BSP method is based on coalescent theory and it is more reflective of the population historical dynamics.

Base on the BSP, our finding showed that the population historical dynamics of *C. taiwaniana* were consistent with the seven *Cycas* species (*C. debaoensis*, *C. simplicipinna*, *C. bifida*, *C. multipinnata*, *C. diannanensis*, *C. segmentifida*, and *C. panzhihuaensis*) which had experienced population contractions based on cpDNA data (Feng et al., 2014, 2017; Gong, 2015; Gong et al., 2015; Liu et al., 2015; Xiao et al., 2020; Zhan et al., 2011). In contrast, the population history dynamics revealed by different nuclear genes were more complex. For nuclear gene *PHYP*, most species had experienced population expansion in history (*C. taiwaniana*, *C. guizhouensis*, and *C. chenii*; Feng, Zheng, & Gong, 2016; Yang et al., 2017). For nuclear gene *PPRC*, *C. taiwaniana* had experienced population expansion, but *C. dolichophylla* had experienced population contractions (Zheng et al., 2016). Avoiding conflicts among different DNA sequences, we performed the EBSP analysis with cpDNA and four nuclear genes using unlinked model. The EBSP result revealed that *C. taiwaniana* had experienced twice population contraction events during the Quaternary period. The Mode shift models showed that the *C. taiwaniana* had not experienced a severe bottleneck in recently, whereas GWIs indicated that this species had experienced bottleneck effect in history, which was similar to the result of EBSP.

Paleobotanical studies suggested that Hainan Island might have been in a much more northerly location with a subtropical Climate during the Eocene (Zhu, 2020). Hainan Island was separated from the Beibu Gulf by rotation from the original position to the current position (counterclockwise 150°), the initial time of separation was in Paleocene (about 65 Mya), and the main period of rotation drift occurred in Eocene (between 40 and 24 Mya; Liang, 2018). The EBSP suggested that *C. taiwaniana* had experienced population contraction events that occurred between Pleistocene Chibanian and Holocene Greenlandian of the Cenozoic Quaternarya (*Ca*. 200 Kya, 10 Kya) and then expansion recently (*Ca*. 7 Kya). During the quaternary period, the glacial and interglacial periods appeared alternately, and the global climate was generally in a state of cooling, but Hainan Island located in the present tropical region was less affected. According to the EBSP result, *C. taiwaniana* was stable at 200 Kya, indicating that the global climate cooling during the glacial period had relatively little influence on *C. taiwaniana* in Hainan Island. During interglacial period (200–15 Kya), the temperature rose, glaciers melted, and sea levels rose, causing *C. taiwaniana* to undergo a population contraction event. The last Great Ice Age occurred about 15 Kya, due to the advent of the ice age, the global climate became colder and the global sea level continued to fall (Liang, 2018). The decline rate of *C. taiwaniana* was weak and experienced a brief population stabilization period. In general, coral reefs grow vigorously, indicating that the temperature rise. According to the development and evolution of coral reefs in Hainan Island, coral reefs flourished about 7300 years ago, indicating that the Holocene great warm period began about 7 Kya, and there were relatively periodic fluctuations of cold–warm, dry–humid (Yan, 2006). According to the latest age of the basalt in Haikou Geopark, the last basalt eruption was about 8 Kya years ago (Liang, 2018), so it was speculated that the volcanic ash might have fallen on Hainan Island to form land. After the last glacial period of late Quaternary and in the early Holocene, the climate of Hainan Island was getting warmer, rainfall increased, and plant growth flourished, *C. taiwaniana* was no exception in increasing its population size until now (Jin et al., 2003, 2008; Wang et al., 2018; Yan, 2006).

### 4.4 Future protection strategy

At present, lots of lives on earth are facing the sixth mass extinction, which is caused by human activities, climate change, and ecological collapse (Teixeira & Huber, 2021). Whether based on DNA data or RAD‐seq data, the genetic diversity of *C. taiwaniana* was relatively low (Tao et al., 2021). Two recent studies on the community structure of *C. taiwaniana* in a certain area revealed that it was a stable population with low growth, but high forest canopy density, habitat destruction, human disturbance and illegal trade might be the reason why *C. taiwaniana* was endangered (Wu et al., 2021; Xie et al., 2019).

Population geneticists usually use empirical assessments of genetic diversity to support one of the core objectives of conservation genetics which is to maintain genetic diversity among individuals in ways that support the sustainability of populations and species, even in the face of continuing threats such as global climate change and fragmentation (Crandall et al., 2000; Moritz, 2002). Among the 12 wild populations of *C. taiwaniana*, the genetic diversity of population DL was the highest and the population NWH had relatively high genetic differentiation with other populations, both of which should be given priority protection. To prevent the loss of genetic diversity or unique genotype, we can use asexual reproduction techniques to maintain genetic diversity within populations, such as bud suctioning.

Effective population size is also an important parameter to evaluate the endangered status of a population or species. In *C. taiwaniana*, the effective population size of populations DLS1, DLS2, and GSL were more than 200 and the other populations were less than 100. The heterozyosity of small populations declined more rapidly than that of large populations, so these populations (BLS, WX, DL, and NBS) might face a high risk of losing partial genetic variation. In addition, 12 wild populations of *C. taiwaniana* were divided into three separated clades, indicating three evolutionary significant units (ESUs) should be managed and protected separately. The three ESUs are ESU1: population NWH; ESU2: population WX, DL, TLF, and NBS; ESU3: population DLS1, DLS2, DLH, DLT, SJC, BLS, and GSL, respectively. Individuals in populations of ESU2 were clumped distribution, which increased inbreeding among them. Inbreeding often occurs when populations have a certain genetic structure. Inbreeding can increase homozygous, leading to the reduction of genetic diversity in small populations, which is extremely dangerous for small groups (Liu & Zhao, 1999). Therefore, combined with results of the estimation of effective population size and the division of significant evolutionary units, appropriate artificial pollination can be implemented in the population BLS and ESU2 to prevent and/or reduce inbreeding. In addition, the genetic diversity of the cultivated population FJ is lower than wild populations, indicating inefficient ex situ conservation. In order to preserve more genetic information, we can select as many individuals (collecting seeds) with high genetic diversity in each population as possible for ex situ protection in the future. Compared with genomic data, the limitations of this study are in existence, and in the future, genomic data will be needed to explore population genetics of more endangered species for formulating more appropriate conservation strategies.

## AUTHOR CONTRIBUTIONS

**Li‐Xin Wu:** Conceptualization (equal); data curation (equal); formal analysis (equal); writing – original draft (equal); writing – review and editing (equal). **Hai‐Yan Xu:** Data curation (equal). **Shu‐Guang Jian:** Investigation (equal); resources (equal). **Xun Gong:** Conceptualization (equal); project administration (lead); supervision (equal); writing – review and editing (equal). **Xiu‐Yan Feng:** Conceptualization (equal); data curation (equal); formal analysis (equal); funding acquisition (lead); project administration (lead); supervision (equal); writing – review and editing (equal).

## CONFLICT OF INTEREST

The authors declare no conflicts of interest.

## Supporting information


Figure S1

Table S1

Table S2

Table S3

Table S4

Table S5

Table S6

Table S7

Table S8

## Data Availability

DNA sequences: GenBank accessions in Table S3. The data that supports the findings of this study are available in the supplementary material of this article.
