# Supplementary figures and images for: Geographic factors and climatic fluctuation drive the genetic structure and demographic history of Cycas taiwaniana (Cycadaceae), an endemic endangered species to Hainan Island in China
Source: Ecol Evol. 2022 Nov 18;12(11):e9508. doi: 10.1002/ece3.9508 (PMC9674470; doi:10.1002/ece3.9508)

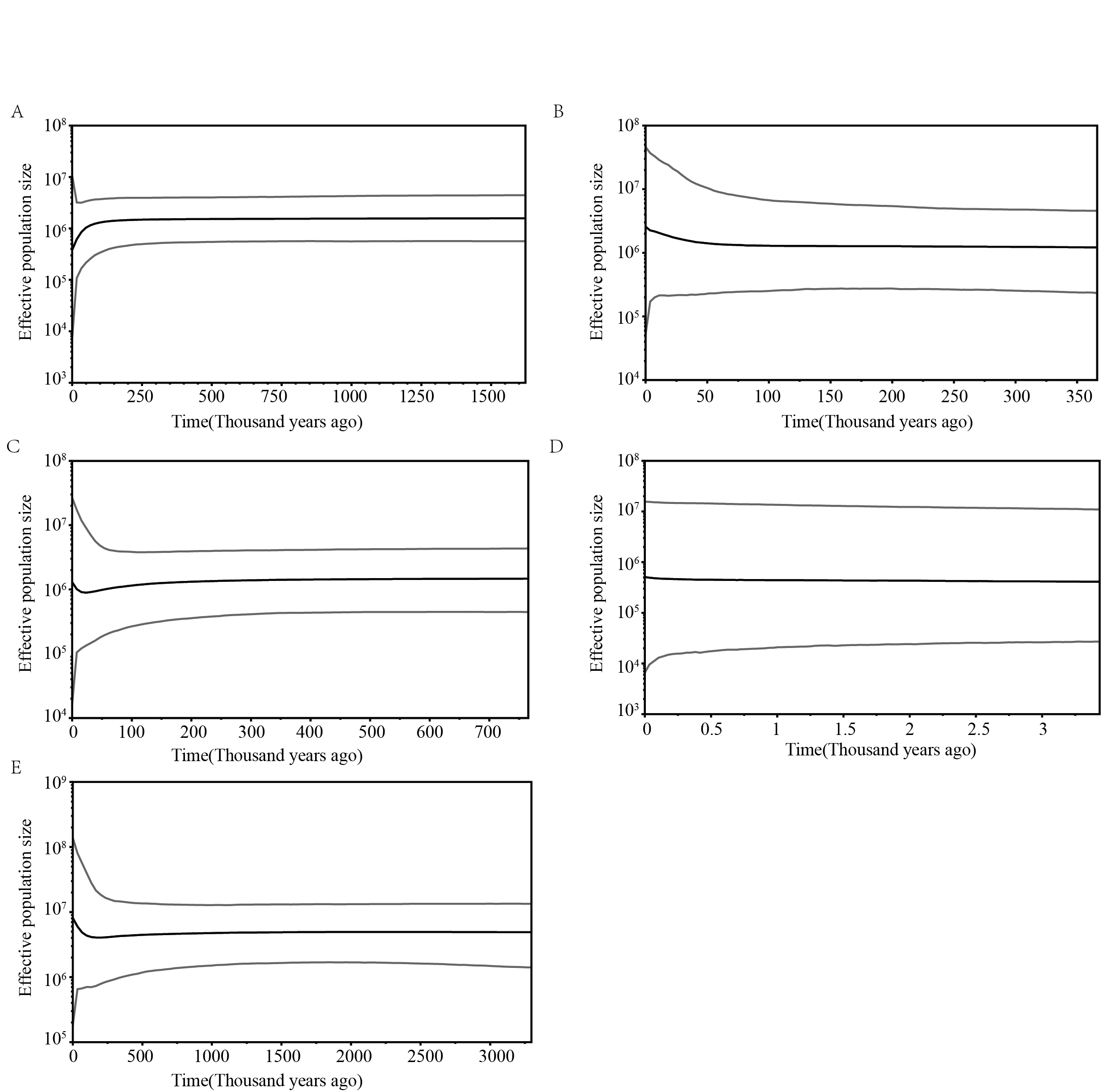

Supplement: Supplementary file 1 — Figure S1 [file ECE3-12-e9508-s002.jpg]
