# Supplementary material for: Geographic factors and climatic fluctuation drive the genetic structure and demographic history of Cycas taiwaniana (Cycadaceae), an endemic endangered species to Hainan Island in China
Source: Ecol Evol. 2022 Nov 18;12(11):e9508. doi: 10.1002/ece3.9508 (PMC9674470; doi:10.1002/ece3.9508)
Supplement: Supplementary file 2 — Table S1 [file ECE3-12-e9508-s006.docx]

Table S1. Details of sample locations, sample sizes surveyed for DNA sequences and microsatellites of *Cycas taiwaniana*

| Population Code | Sample Sites | Latitude  N° | Longitude  E° | Individuals for DNA sequences/Microsatellites |
| --- | --- | --- | --- | --- |
| DLS1 | Diaoluoshan, Hainan | 18.783 | 109.833 | 10/20 |
| DLS2 | Diaoluoshan, Hainan | 18.733 | 109.783 | 10/20 |
| DLH | Diaoluoshan, Hainan | 18.867 | 109.580 | 10/20 |
| DLT | Diaoluoshan, Hainan | 18.914 | 109.658 | 9/9 |
| SJC | Shenjiecun, Qiongzhong, Hainan | 18.900 | 109.917 | 10/12 |
| BLS | Baolongshan, Hainan | 18.583 | 109.433 | 10/11 |
| GSL | Ganshenling, Hainan | 18.500 | 109.650 | 10/20 |
| NWH | Nanwanhou, Hainan; | 19.300 | 109.850 | 10/20 |
| FJ | Fujian | 24.117 | 117.167 | 9/9 |
| WX | Wangxia, Hainan | 19.000 | 109.117 | 10/12 |
| DL | Dongliu, Hainan | 19.117 | 109.200 | 10/12 |
| TLF | Tuolingfeng, Hainan | 19.050 | 109.233 | 10/12 |
| NBS | Nanbaoshan, Hainan | 19.133 | 109.417 | 10/12 |
| Total |  |  |  | 128/188 |
