# Supplementary material for: Geographic factors and climatic fluctuation drive the genetic structure and demographic history of Cycas taiwaniana (Cycadaceae), an endemic endangered species to Hainan Island in China
Source: Ecol Evol. 2022 Nov 18;12(11):e9508. doi: 10.1002/ece3.9508 (PMC9674470; doi:10.1002/ece3.9508)
Supplement: Supplementary file 3 — Table S2 [file ECE3-12-e9508-s009.docx]

Table S2. Information of cpDNA intergenic spacers, nuclear genes DNA and microsatellites used in this study

| Primers | Primer sequences (5′→3′) | Repeat motif | Reference |
| --- | --- | --- | --- |
| *atp*B-*rbc*L | *atp*B: ACATCKARTACKGGACCAATAA  *rbc*L: AACACCAGCTTTRAATCCAA | — | (Sang et al., 1997) |
| *psb*A-*trn*H | *psb*A: GTTATGCATGAACGTAATGCTC  *trn*H: CGCGCATGGTGGATTCACAAATC | — | (Chiang & Peng, 1998) |
| *psb*M-*trn*D | *psb*M: AGCAATAAATGCRAGAATATTTACTTCCAT  *trn*D: GGGATTGTAGYTCAATTGGT | — | (Shaw et al., 2005) |
| *trn*S*-trn*G | *trn*S: GCCGCTTTAGTCCACTCAGC  *trn*G: GAACGAATCACACTTTTACCAC | — | (Shaw et al., 2005) |
| *AC*5 | *AC*5-F: ATGCGCCGAGGGCGGTGTTC  *AC*5-R: AGATGCGAGGATGGAGCCTC | — | (Feng et al., 2021) |
| *PHYP* | *PHYP*-F: CCAGTCTCCCAGTATCATGG  *PHYP*-R: GCTGCATGATATTTCCAACC | — | (Feng et al., 2016) |
| *PPRC* | *PPRC*-F: CAAAACTATGCTGTCAATCC  *PPRC*-R: TTAGCATCACCAGTAATCCC | — | (Feng et al., 2017) |
| *AAT* | *AAT*-F: CACAGCTGGAAGCACATCAC  *AAT*-R: GCACTTTTAGCATGGAAGGC | — | (Feng et al., 2021) |
| Cha02 | F: CGAGGAACATCAAGGCTATG  R: CCTAGCTTTTGGGAATTAGAC | (CT)_21_ | (Zhang et al., 2009) |
| Cha05 | F: GTCTGCTAACATCTATAAA  R: GATGAGCTAAGAGTCATAGTA | (CT)_19_ | (Zhang et al., 2009) |
| Cha08 | F: CAGGGACCATTGTTTCTAAGG  R: ACTTATACATAGGGCTCTAAT | (AG)_10_ | (Zhang et al., 2009) |
| Cy-Tai EST-SSR11 | F: GATATTAAAGGCACGGGAG  R: TGAAGCTGCTGCATTTGCAT | (CAG)_34_ | (Ju et al., 2011) |
| E001 | F: TGGGATTAATATTCCAGAAA  R: CGACGAGTCTGATGTAGGTAT | (CA)_10_ | (Yang et al., 2008) |
| E004 | F: CTATCATCAGAGCCTCATTC  R: AAGTCATACATGGACAGCAA | (AT)_11_ | (Yang et al., 2008) |
| HL03 | F: GAGATAGGCTTGGAAGCTTAT  R: GTCATAGCCCTCTCTAACAT | (AG)_23_ | (Li et al., 2009) |
| HL08 | F: AAAACATTCCTTGCCCTGT  R: GGAGCCTGTTGAAGAGCTA | (TTC)_12_ | (Li et al., 2009) |
| Cha-estssr02 | F: ATAGGCTTCCTTTAGTGATGTC  R: GCCTTTAGTAGTATCGGATTA | (CT)_5_(AG)_4_G(GA)_5_ | (Wang et al., 2008) |
| Cha-estssr04 | F: GATGTTCCCAAATAATGTTACA  R: CAAGCTGCACATGCAATGA | (AT)_3_GT(AT)_9_AG(AC)_4_ | (Wang et al., 2008) |

**Literature Cited**

Chiang, T.Y., Peng, C.I., 1998. Phylogeography of the endemic plants in Taiwan in: Yeng, S.D. (ed.). Pages 148–155. Proceeding of the Symposium on Conservation of Endemic Species. Research Institute of Taiwan Endemic Species, Taipei.

Feng, X.Y., Liu, J., Chiang, Y.C., Gong, X., 2017. Investigating the Genetic Diversity, Population Differentiation and Population Dynamics of *Cycas segmentifida* (Cycadaceae) Endemic to Southwest China by Multiple Molecular Markers. Front. Plant. Sci. 8: 839.

Feng, X.Y., Liu, J., Gong, X., 2016. Species Delimitation of the *Cycas segmentifida* Complex (Cycadaceae) Resolved by Phylogenetic and Distance Analyses of Molecular Data. Front. Plant. Sci. 7: 134.

Feng, X.Y., Wang, X.H., Chiang, Y.C., Jian, S.G., Gong, X., 2021. Species delimitation with distinct methods based on molecular data to elucidate species boundaries in the *Cycas taiwaniana* complex (Cycadaceae). Taxon 70(3): 477–491.

Ju, L.P., Kuo, C.C., Chao, Y.S., Cheng, Y.P., Gong, X., Chiang, Y.C., 2011. Microsatellite primers in the native perennial cycad *Cycas taitungensis* (Cycadaceae). Am. J. Bo. 98(4):e84-86.

Li, L., Wang, Z.F., Jian, S.G., Zhu, P., Zhang, M., Ye, W.H., Ren, H., 2009. Isolation and characterization of microsatellite loci in endangered *Cycas changjiangensis* (Cycadaceae). Conserv. Genet. 10(3):793-795.

Sang, T., Crawford, D., Stuessy, T., 1997. Chloroplast DNA phylogeny, reticulate evolution, and biogeography of Paeonia (Paeoniaceae). Am. J. Bot. 84(9):1120.

Shaw, J., Lickey, EB., Beck, J.T., Farmer, S.B., Liu, W., Miller, J., Siripun, K.C., Winder, C.T., Schilling, E.E., Small, R.L., 2005. The tortoise and the hare II: relative utility of 21 noncoding chloroplast DNA sequences for phylogenetic analysis. Am. J. Bot. 92(1):142-166.

Wang, Z.F., Ye, W.H., Cao, H.L., Li, Z.C., Peng, S.L., 2008. Identification and characterization of EST-SSRs and cpSSRs in endangered *Cycas hainanensis*. Conserv. Genet. 9:1079-1081.

Yang, Y., Li, Y., Li, L.F., Ge, X.J., Gong, X., 2008. Isolation and characterization of microsatellite markers for *Cycas debaoensis* Y. C. Zhong et C. J. Chen (Cycadaceae). Mol. Ecol. Resour. 8:913-915.

Zhang, M., Wang, Z.F., Jian, S.G., Ye, W.H., Cao, H.L., Zhu, P., Li, L., 2009. Isolation and characterization of microsatellite markers for *Cycas hainanensis* C. J. Chen (Cycadaceae). Conserv. Genet. 10:1175–1176.
