# Supplementary material for: Geographic factors and climatic fluctuation drive the genetic structure and demographic history of Cycas taiwaniana (Cycadaceae), an endemic endangered species to Hainan Island in China
Source: Ecol Evol. 2022 Nov 18;12(11):e9508. doi: 10.1002/ece3.9508 (PMC9674470; doi:10.1002/ece3.9508)
Supplement: Supplementary file 4 — Table S3 [file ECE3-12-e9508-s001.docx]

Table S3. GenBank accession numbers of sequences used for population genetics analysis in this study

| pop | DLS1 | DLS2 | BLS | GSL | NWH | SJC | DLH | FJ | DLT | WX | DL | TLF | NBS |
| --- | --- | --- | --- | --- | --- | --- | --- | --- | --- | --- | --- | --- | --- |
| atpB-rbcL | HapB1 | HapB1 | HapB1 | HapB1 | HapB2 | HapB1 | HapB1 & HapB3 | HapB1 | HapB1 | HapB4 | HapB5 | HapB6 | HapB6 |
|  | MN539692 | MN539692 | MN539692 | MN539692 | MN539693 | MN539692 | MN539692 & MN539694 | MN539692 | MN539692 | MN539695 | MN539696 | MN539697 | MN539697 |
| *psb*A-*trn*H | HapA1 | HapA1 | HapA1 | HapA1 | HapA1 | HapA1 | HapA2 | HapA1 | HapA3 | HapA4 | HapA1 | HapA4 | HapA4 |
|  | MN539673 | MN539673 | MN539673 | MN539673 | MN539673 | MN539673 | MN539674 | MN539673 | MN539675 | MN539676 | MN539673 | MN539676 | MN539676 |
| *psb*M-*trn*D | HapM1 | HapM1 | HapM2 | HapM2 | HapM1 | HapM1 | HapM1 | HapM1 | HapM1 | HapM1 | HapM3 | HapM1 | HapM1 |
|  | MN539679 | MN539679 | MN539680 | MN539680 | MN539679 | MN539679 | MN539679 | MN539679 | MN539679 | MN539679 | MN539681 | MN539679 | MN539679 |
| *trn*S-*trn*G | HapS1 | HapS1 | HapS2 | HapS2 & taiS3 | HapS1 | HapS1 | HapS1 | HapS1 | HapS1 | HapS3 | HapS4 | HapS5 | HapS6 |
|  | MN539684 | MN539684 | MN539685 | MN539685 & OM902635 | MN539684 | MN539684 | MN539684 | MN539684 | MN539684 | MN539686 | MN539687 | MN539688 | MN539689 |
| AC5 | taiA1-5 | taiA1-3&5-6 | taiA1,3,5 | taiA1,3-5,7 | taiA1,8 | taiA1,5,9 | taiA1,3,6,10 | taiA1 | taiA1-3,5,11 | taiA1,3-5,12-15 | taiA3,4,15,16 | taiA1,3,4,17 | taiA1,3,4,17 |
|  | MW207175, MW207178, MW207177, MW207187, MW207176 | MW207175, MW207178, MW207177, MW207176, MW207179 | MW207175, MW207177, MW207176 | MW207175, MW207177, MW207187, MW207176, OM902636 | MW207175, MW207181 | MW207175, MW207176, OM902637 | MW207175, MW207177, MW207179, MW207183 | MW207175 | MW207175, MW207178, MW207177, MW207176, MW207184 | MW207175, MW207177, MW207187, MW207176, MW207185, MW207186, OM902638, MW207190 | MW207177, MW207187, MW207190, MW207191 | MW207175, MW207177, MW207187, MW207189 | MW207175, MW207177, MW207187, MW207189 |
| PHYP | taiP1-3 | taiP1,2,4 | taiP1,2 | taiP1,2 | taiP1,2,4-6 | taiP1,2,4 | taiP1,2,4,7,8 | taiP1,5 | taiP1,2,5,9 | taiP1,2,5,10-12 | taiP1,2,5,6,10 | taiP1,2,6,10 | taiP1,2,5,6,10 |
|  | OM902639, OM902640, OM902641 | OM902639, OM902640, OM902642 | OM902639, OM902640 | OM902639, OM902640 | OM902639, OM902640, OM902642, OM902643, OM902644 | OM902639, OM902640, OM902642 | OM902639, OM902640, OM902642, OM902645, OM902646 | OM902639, OM902643 | OM902639, OM902640, OM902643, OM902647 | OM902639, OM902640, OM902643, OM902648, OM902649, OM902650 | OM902639, OM902640, OM902643, OM902644, OM902648 | OM902639, OM902640, OM902644, OM902648 | OM902639, OM902640, OM902643, OM902644, OM902648 |
| PPRC | taiR1-3 | taiR1,2,4 | taiR1,2 | taiR1,2 | taiR1,2 | taiR1-3,5 | taiR1,2,5 | taiR2,6 | taiR1,2 | taiR1,2,7-9 | taiR1,2,10,11 | taiR1,2,10 | taiR1,2,10 |
|  | MW219054, MW219055, MW219056 | MW219054, MW219055, OM902651 | MW219054, MW219055 | MW219054, MW219055 | MW219054, MW219055 | MW219054, MW219055, MW219056, OM902652 | MW219054, MW219055, OM902652 | MW219055, MW219062 | MW219054, MW219055 | MW219054, MW219055, MW219058, MW219059, OM902653 | MW219054, MW219055, MW219060, MW219061 | MW219054, MW219055, MW219060 | MW219054, MW219055, MW219060 |
| AAT | taiT1-10 | taiT1,5-7,11-13 | taiT5-7,14-16 | taiT3,5,6,12,14,17 | taiT3,5,6,12,13,18-21 | taiT1,3,5,6,22,23 | taiT1,5,6,14,15,20 | taiT3,24 | taiT1,5,6,23,25 | taiT6,14,25-30 | taiT5,6,9,12,17,27,29,31-36 | taiT5,12,14,24,33,34,37-40 | taiT6,9,14,36,41-43 |
|  | MW219065, OM902654, MW219075, OM902655, MW219067, MW219066, MW219068, MW219069, MW219087,  OM902656 | MW219065, MW219067, MW219066, MW219068, MW219070, MW219074, MW219078 | MW219067, MW219066, MW219068, MW219071, MW219072, OM902657 | MW219075, MW219067, MW219066, MW219074, MW219071, MW219073 | MW219075, MW219067, MW219066, MW219074, MW219078, MW219076, OM902658, OM902659, MW219077 | MW219065, MW219075, MW219067, MW219066, OM902660, MW219079 | MW219065, MW219067, MW219066, MW219071, MW219072, OM902659 | MW219075, MW219080 | MW219065, MW219067, MW219066, MW219079, MW219081 | MW219066, MW219071, MW219081, MW219082, MW219085, OM902661, MW219083, MW219084 | MW219067, MW219066, MW219087, MW219074, MW219073, MW219085, MW219083, MW219086, MW219088, MW219093, MW219091, MW219089, MW219094 | MW219067, MW219074, MW219071, MW219080, MW219093, MW219091, MW219090, MW219092, OM902662, OM902663 | MW219066, MW219087, MW219071, MW219094, OM902664, OM902665, MW219095 |
