# Supplementary material for: Geographic factors and climatic fluctuation drive the genetic structure and demographic history of Cycas taiwaniana (Cycadaceae), an endemic endangered species to Hainan Island in China
Source: Ecol Evol. 2022 Nov 18;12(11):e9508. doi: 10.1002/ece3.9508 (PMC9674470; doi:10.1002/ece3.9508)
Supplement: Supplementary file 5 — Table S4 [file ECE3-12-e9508-s004.docx]

Table S4. Composition of haplotypes in populations of *Cycas taiwaniana* derived from combined plastid DNA and nuclear genes.

| Population code | cpDNA | *AC*5 | *PHYP* | | *PPRC* | | *AAT* |
| --- | --- | --- | --- | --- | --- | --- | --- |
|  | Haplotypes (No.) | Haplotypes (No.) | | Haplotypes (No.) | | Haplotypes (No.) | Haplotypes (No.) |
| DLS1 | taiH1(10) | taiA1(11) A2(1) A3(4) A4(2) A5(2) | | taiP1(13) P2(6) P3(1) | | taiR1(5) R2(14) R3(1) | taiT1(5) T2-T4(1) T5(3) T6(5) T7-T10(1) |
| DLS2 | taiH1(10) | taiA1(2) A2(2) A3(8) A5(6) A6(2) | | taiP1(12) P2(7) P4(1) | | taiR1(7) R2(12) R4(1) | taiT1(3) T5(8) T6(4) T7(1) T11(2) T12(1) T13(1) |
| DLH | taiH5(8) taiH6(2) | taiA1(8) A3(10) A6(1) A10(1) | | taiP1(7) P2(6) P4(5) P7(1) P8(1) | | taiR1(6) R2(13) R5(1) | taiT1(2) T5(4) T6(7) T14(2) T15(4) T20(1) |
| DLT | taiH7(9) | taiA1(7) A2(1) A3(5) A5(4) A11(1) | | taiP1(9) P2(6) P5(2) P9(1) | | taiR1(7) R2(11) | taiT1(1) T5(5) T6(10) T23(1) T25(1) |
| SJC | taiH1(10) | taiA1(16) A5(1) A9(3) | | taiP1(15) P2(4) P4(1) | | taiR1(4) R2(9) R3(4) R5(3) | taiT1(2) T3(2) T5(8) T6(3) T22(2) T23(3) |
| BLS | taiH2(10) | taiA1(7) A3(7) A5(6) | | taiP1(15) P2(5) | | taiR1(3) R2(17) | TaiT5(4) T6(9) T7(1) T14(4) T15(1) T16(1) |
| GSL | taiH2(9) taiH3(1) | taiA1(5) A3(8) A4(1) A5(5) A7(1) | | taiP1(11) P2(9) | | taiR1(6) R2(14) | TaiT3(1) T5(6) T6(6) T12(1) T14(4) T17(2) |
| NWH | taiH4(10) | taiA1(9) A8(11) | | taiP1(2) P2(5) P4(7) P5(4) P6(2) | | taiR1(11) R2(9) | TaiT3(1) T5(4) T6(5) T12(1) T13(1) T18(3) T19(3) T20(1) T21(1) |
| FJ | taiH1(9) | taiA1(18) | | taiP1(9) P5(9) | | taiR2(9) R6(9) | TaiT3(9) T24(9) |
| WX | taiH8(10) | taiA1(1) A3(2) A4(5) A5(7) A12(1) A13(1) A14(2) A15(1) | | taiP1(1) P2(1) P5(1) P10(8) P11(8) P12(1) | | taiR1(1) R2(2) R7(13) R8(3) R9(1) | taiT6(8) T14(1) T25(2) T26(3) T27(3) T28-T30(1) |
| DL | taiH9(10) | taiA3(14) A4(2) A15(1) A16(3) | | taiP1(2) P2(4) P5(8) P6(4) P10(2) | | TaiR1(1)R2(14) R10(2) R11(3) | taiT5(1) T6(1) T9(1) T12(2) T17(1) T27(3) T29(2) T31(3) T32(1) T33(1) T34(2) T35(1) T36(1) |
| TLF | taiH10(10) | taiA1(4) A3(11) A4(3) A17(2) | | taiP1(11) P2(5) P6(3) P10(1) | | taiR1(14) R2(4) R10(2) | taiT5(1) T12(1) T14(2) T24(2) T33(3) T34(6) T37(1) T38(2) T39(1) T40(1) |
| NBS | taiH11(10) | taiA1(3) A3(11) A4(5) A17(1) | | taiP1(3) P2(4) P5(7) P6(1) P10(5) | | taiR1(4) R2(14) R10(2) | taiT6(4) T9(1) T14(8) T36(3) T41(1) T42(2) T43(1) |
| Total | 11 | 17 | | 12 | | 11 | 43 |
