# Supplementary material for: Geographic factors and climatic fluctuation drive the genetic structure and demographic history of Cycas taiwaniana (Cycadaceae), an endemic endangered species to Hainan Island in China
Source: Ecol Evol. 2022 Nov 18;12(11):e9508. doi: 10.1002/ece3.9508 (PMC9674470; doi:10.1002/ece3.9508)
Supplement: Supplementary file 6 — Table S5 [file ECE3-12-e9508-s005.docx]

Table S5. Summary of the ten SSR loci used to study the population genetics of *Cycas taiwaniana*

| Locus | *A*_R_ | *N*_A_ | *N*_E_ | *I* | *H*_O_ | *H*_E_ | *UHE* | *F*_IS_ | *F*_IT_ | *F*_ST_ | *H*s | *H*_T_ | *G*_ST_ | *G’*_ST_ | *Nm* |
| --- | --- | --- | --- | --- | --- | --- | --- | --- | --- | --- | --- | --- | --- | --- | --- |
| Cha02 | 6.215 | 16 | 4.700 | 1.596 | 0.643 | 0.710 | 0.736 | 0.094 | 0.297 | 0.224 | 0.741 | 0.916 | 0.191 | 0.204 | 0.868 |
| Cha05 | 4.815 | 16 | 3.331 | 1.281 | 0.547 | 0.624 | 0.648 | 0.124 | 0.306 | 0.208 | 0.653 | 0.790 | 0.174 | 0.186 | 0.952 |
| Cha08 | 5.546 | 23 | 3.763 | 1.450 | 0.708 | 0.688 | 0.716 | -0.028 | 0.129 | 0.154 | 0.716 | 0.815 | 0.122 | 0.131 | 1.378 |
| Cy-TaiEST-SSR11 | 1.517 | 4 | 1.071 | 0.133 | 0.059 | 0.063 | 0.065 | 0.064 | 0.091 | 0.029 | 0.066 | 0.065 | -0.010 | -0.011 | 8.473 |
| E001 | 2.829 | 5 | 2.137 | 0.847 | 0.568 | 0.516 | 0.537 | -0.099 | 0.014 | 0.103 | 0.535 | 0.577 | 0.072 | 0.077 | 2.187 |
| E004 | 4.927 | 16 | 3.366 | 1.320 | 0.236 | 0.653 | 0.679 | 0.640 | 0.690 | 0.140 | 0.697 | 0.763 | 0.086 | 0.093 | 1.540 |
| HL03 | 5.057 | 12 | 3.189 | 1.337 | 0.661 | 0.651 | 0.676 | -0.016 | 0.187 | 0.200 | 0.677 | 0.815 | 0.170 | 0.182 | 0.999 |
| HL08 | 5.158 | 12 | 3.543 | 1.408 | 0.725 | 0.687 | 0.714 | -0.056 | 0.106 | 0.153 | 0.713 | 0.813 | 0.122 | 0.131 | 1.385 |
| Cha-estssr02 | 2.075 | 6 | 1.484 | 0.427 | 0.125 | 0.248 | 0.258 | 0.495 | 0.776 | 0.556 | 0.263 | 0.560 | 0.530 | 0.550 | 0.199 |
| Cha-estssr04 | 4.210 | 10 | 2.827 | 1.414 | 0.382 | 0.584 | 0.606 | 0.346 | 0.504 | 0.242 | 0.616 | 0.773 | 0.202 | 0.216 | 0.785 |
| Mean | 4.235 | 12 | 2.941 | 1.094 | 0.465 | 0.542 | 0.563 | 0.156 | 0.310 | 0.201 | 0.568 | 0.689 | 0.176 | 0.188 | 1.877 |
| Total |  | 120 |  |  |  |  |  |  |  |  |  |  |  |  |  |

Note: *A*_R_, allelic richness; *N*_A_, number of alleles; *N*_E_, the effective number of alleles; *I*, Shannon’s information index; *H*_O_, observed heterozygosity; *H*_E_, expected heterozygosity; *UHE*, Nei’s unbiased heterozygosity; *F*, fixation index (*F*_IS_, *F*_IT_, *F*_ST_); *Nm*, gene flow
