# Supplementary material for: Geographic factors and climatic fluctuation drive the genetic structure and demographic history of Cycas taiwaniana (Cycadaceae), an endemic endangered species to Hainan Island in China
Source: Ecol Evol. 2022 Nov 18;12(11):e9508. doi: 10.1002/ece3.9508 (PMC9674470; doi:10.1002/ece3.9508)
Supplement: Supplementary file 7 — Table S6 [file ECE3-12-e9508-s008.docx]

Table S6. Gene flow between each pair of 13 populations of *Cycas taiwaniana* based on the SSR data

| Population | DLS1 | DLS2 | DLH | DLT | SJC | BLS | GSL | NWH | FJ | WX | DL | TLF | NBS |
| --- | --- | --- | --- | --- | --- | --- | --- | --- | --- | --- | --- | --- | --- |
| DLS1 | 0.000 |  |  |  |  |  |  |  |  |  |  |  |  |
| DLS2 | 8.452 | 0.000 |  |  |  |  |  |  |  |  |  |  |  |
| DLH | 4.228 | 5.353 | 0.000 |  |  |  |  |  |  |  |  |  |  |
| DLT | 3.967 | 5.459 | 5.453 | 0.000 |  |  |  |  |  |  |  |  |  |
| SJC | 3.206 | 2.486 | 1.982 | 2.596 | 0.000 |  |  |  |  |  |  |  |  |
| BLS | 3.260 | 4.627 | 3.011 | 6.164 | 3.723 | 0.000 |  |  |  |  |  |  |  |
| GSL | 3.181 | 4.268 | 2.652 | 3.332 | 2.201 | 6.982 | 0.000 |  |  |  |  |  |  |
| NWH | 0.856 | 0.781 | 1.088 | 0.922 | 1.016 | 0.769 | 0.657 | 0.000 |  |  |  |  |  |
| FJ | 0.902 | 0.994 | 1.200 | 1.028 | 0.907 | 1.272 | 1.135 | 0.481 | 0.000 |  |  |  |  |
| WX | 0.616 | 0.664 | 0.761 | 0.596 | 0.578 | 0.607 | 0.463 | 0.625 | 0.349 | 0.000 |  |  |  |
| DL | 1.543 | 1.392 | 1.840 | 1.128 | 1.361 | 1.091 | 1.035 | 0.984 | 0.699 | 1.099 | 0.000 |  |  |
| TLF | 1.304 | 1.369 | 1.554 | 1.303 | 1.171 | 1.014 | 0.991 | 1.158 | 0.652 | 1.221 | 3.479 | 0.000 |  |
| NBS | 0.670 | 0.684 | 0.663 | 0.531 | 0.546 | 0.510 | 0.507 | 0.499 | 0.330 | 0.759 | 1.636 | 1.509 | 0.000 |
