# Supplementary material for: Geographic factors and climatic fluctuation drive the genetic structure and demographic history of Cycas taiwaniana (Cycadaceae), an endemic endangered species to Hainan Island in China
Source: Ecol Evol. 2022 Nov 18;12(11):e9508. doi: 10.1002/ece3.9508 (PMC9674470; doi:10.1002/ece3.9508)
Supplement: Supplementary file 8 — Table S7 [file ECE3-12-e9508-s007.docx]

Table S7. Genetic differentiation parameters for 12 wild population of *Cycas taiwaniana* based on the combined cpDNA sequence and four nuclear genes.

| Markers | *G*_ST_ | *N*_ST_ | *P* |
| --- | --- | --- | --- |
| cpDNA | 0.951 | 0.968 | 0.171^ns^ |
| *AC*5 | 0.125 | 0.157 | 0.248^ns^ |
| *AAT* | 0.078 | 0.148 | 0.002^*^ |
| *PHYP* | 0.160 | 0.283 | 0.033^*^ |
| *PPRC* | 0.176 | 0.186 | 0.331^ns^ |

Note: ^ns^, not significant; ^*^, *P*＜0.05; ^**^, *P*＜0.01; ^***^, *P* ＜0.001.
