# Supplementary material for: Geographic factors and climatic fluctuation drive the genetic structure and demographic history of Cycas taiwaniana (Cycadaceae), an endemic endangered species to Hainan Island in China
Source: Ecol Evol. 2022 Nov 18;12(11):e9508. doi: 10.1002/ece3.9508 (PMC9674470; doi:10.1002/ece3.9508)
Supplement: Supplementary file 9 — Table S8 [file ECE3-12-e9508-s003.docx]

Table S8. Parameters of neutrality tests and mismatch distribution analysis based on cpDNA and nuclear genes of *Cycas taiwaniana*

| Marker | Tajima’*D* | Fu and Li’*D** | Fu and Li’*F** | Fu’s *Fs* | SSD | raggedness |
| --- | --- | --- | --- | --- | --- | --- |
| cpDNA | -1.2528 | -2.7955* | -2.6650* | -0.2070 | 0.0194* | 0.1157 |
| *AC5* | -0.6962 | 0.3073 | -0.0941 | -5.0930* | 0.0833 | 0.1108 |
| *PHYP* | 0.1630 | 0.6210 | 0.5418 | 0.5418 | 0.0395 | 0.1786 |
| *PPRC* | -1.4675 | 0.4776 | -0.3391 | -1.9250 | 0.0458 | 0.1833 |
| *AAT* | -0.1352 | 1.1640 | 0.7486 | -18.5660* | 0.0426 | 0.0900 |

Note: *, P<0.05
